# Supplementary material for: Single-cell analysis of hepatoblastoma identifies tumor signatures that predict chemotherapy susceptibility using patient-specific tumor spheroids
Source: Nat Commun. 2022 Aug 25;13:4878. doi: 10.1038/s41467-022-32473-z (PMC9411569; doi:10.1038/s41467-022-32473-z)
Supplement: Supplementary file 3 — Description of Additional Supplementary Files [file 41467_2022_32473_MOESM3_ESM.pdf]

Description of Additional Supplementary Files

File Name: Supplementary Data 1
Description: De-identified clinical information of the nine HB patients in the study.

File Name: Supplementary Data 2
Description: Single-cell RNA sequencing cell quality summary and annotation information. Numbers of cells captured, mean and median numbers of genes/UMIs, and mean percentage of mitochondrial gene expressions are shown for each sequenced array. Manual annotations are validated and consistent with automated annotation by SingleR. Signature gene sets generated from manual annotation are specifically listed.

File Name: Supplementary Data 3
Description: Top 50 differentially expressed genes in each tumor cell cluster. Gene symbols, average expression log2 fold change, and adjusted p-values are shown.

File Name: Supplementary Data 4
Description: Differentially expressed gene sets of the five HB Signatures. Gene symbols, average expression log2 fold change, and adjusted p-values are shown.

File Name: Supplementary Data 5
Description: Origin of cultivated Patientderived spheroids (Freshly isolated cells or Cryopreserved freshly isolated cells) and ability of cells to grow at short term (2- 4 passages) or long term (more than 16 passages).

File Name: Supplementary Data 6
Description: Differentially expressed genes in each Patient-derived spheroids lines. Gene symbols, pvalues, average expression log2 fold change, Pct1, Pct2 and adjusted pvalues are shown.
