# Supplementary material for: Single-cell analysis of hepatoblastoma identifies tumor signatures that predict chemotherapy susceptibility using patient-specific tumor spheroids
Source: Nat Commun. 2022 Aug 25;13:4878. doi: 10.1038/s41467-022-32473-z (PMC9411569; doi:10.1038/s41467-022-32473-z)
Supplement: Supplementary file 10 — Reporting Summary [file 41467_2022_32473_MOESM10_ESM.pdf]

nature portfolio

Corresponding author(s): Bruce Wang, Amar Nijagal, and Franklin Huang

Last updated by author(s): Jul 14, 2022

Reporting Summary

Nature Portfolio wishes to improve the reproducibility of the work that we publish. This form provides structure for consistency and transparency in reporting. For further information on Nature Portfolio policies, see our Editorial Policies and the Editorial Policy Checklist.

Statistics

For all statistical analyses, confirm that the following items are present in the figure legend, table legend, main text, or Methods section.

n/a	Confirmed	
☐	☒	The exact sample size (*n*) for each experimental group/condition, given as a discrete number and unit of measurement
☐	☒	A statement on whether measurements were taken from distinct samples or whether the same sample was measured repeatedly
☐	☒	The statistical test(s) used AND whether they are one- or two-sided<br>*Only common tests should be described solely by name; describe more complex techniques in the Methods section.*
☒	☐	A description of all covariates tested
☒	☐	A description of any assumptions or corrections, such as tests of normality and adjustment for multiple comparisons
☐	☒	A full description of the statistical parameters including central tendency (e.g. means) or other basic estimates (e.g. regression coefficient) AND variation (e.g. standard deviation) or associated estimates of uncertainty (e.g. confidence intervals)
☒	☐	For null hypothesis testing, the test statistic (e.g. *F*, *t*, *r*) with confidence intervals, effect sizes, degrees of freedom and *P* value noted<br>*Give P values as exact values whenever suitable.*
☒	☐	For Bayesian analysis, information on the choice of priors and Markov chain Monte Carlo settings
☒	☐	For hierarchical and complex designs, identification of the appropriate level for tests and full reporting of outcomes
☒	☐	Estimates of effect sizes (e.g. Cohen's *d*, Pearson's *r*), indicating how they were calculated

Our web collection on statistics for biologists contains articles on many of the points above.

Software and code

Policy information about availability of computer code

Data collection	Sequencing results were returned as paired FASTQ reads and processed with FastQC (v0.11.9) for general quality checks in order to further improve our experimental protocol. Then, the paired FASTQ files were aligned against the reference genome using a STAR aligner in the dropseq workflow (https://cumulus.readthedocs.io/en/latest/drop_seq.html). The aligning pipeline output included aligned and corrected bam files, two digital gene expression (DGE) matrix text files (a raw read count matrix and a UMI-collapsed read count matrix where multiple reads that matched the same UMI would be collapsed into one single UMI count) and text-file reports of basic sample qualities such as the number of beads used in the sequencing run, total number of reads, alignment logs.
Data analysis	Sequencing and alignment<br>Sequencing results were returned as paired FASTQ reads and the paired FASTQ files were aligned against hg19 reference genome (GRCh37.p13) using the dropseq workflow (https://cumulus.readthedocs.io/en/latest/drop_seq.html). The aligning pipeline output included aligned and corrected bam files, two digital gene expression (DGE) matrix text files (a raw read count matrix and a UMI-collapsed read count matrix where multiple reads that matched the same UMI would be collapsed into one single UMI count) and text-file reports of basic sample qualities such as the number of beads used in the sequencing run, total number of reads, alignment logs. For each sample, the median and average number of genes per barcode were 1,014 and 1,224. The median and average number of UMI were 2,536 and 3,974. The mean percentage of mitochondrial content per cell was 17.06%.<br><br>Single-cell clustering analysis<br>Cells captured in single-cell RNA sequencing analysis were clustered and analyzed using customized codes based on the Seurat (Version 3.2) package in R (Version 4.0.3). Cells with fewer than 300 genes, 500 transcripts, or a mitochondrial level of 20% or greater, were filtered out as the first QC process. Then, by examining the distribution histogram of the number of genes per cell in each sample, we set the upper threshold for the number of genes per cell in each individual sample in order to filter potential doublets. A total of 29,968 cells were acquired using these thresholds.<br>UMI-collapsed read-count matrices for each cell were loaded in Seurat for analysis. We followed a standard workflow by using the

"LogNormalize" method that normalized the gene expression for each cell by the total expression, multiplying by a scale factor 10,000. For downstream analysis to identify different cell types, we then calculated and returned the top 2,000 most variably expressed genes among the cells before applying a linear scaling by shifting the expression of each gene in the dataset so that the mean expression across cells was 0 and the variance was 1. This way, the gene expression level could be comparable among different cells and genes. Principal components analysis (PCA) was run using the previously determined most variably expressed genes for linear dimensional reduction and the first 100 principal components (PCs) were stored, which accounted for 40.49% of the total variance. To determine how many PCs to use for the clustering, a JackStraw resampling method was implemented by permutation on a subset of data (1% by default) and rerunning PCA for a total of 100 replications to select the statistically significant PC to include for the K-nearest neighbors clustering. For graph-based clustering, the first 75 PC and a resolution of 1.2 were selected, yielding 37 cell clusters. We eliminated the clustering side effect due to over clustering by constructing a cluster tree of the average expression profile in each cluster and merging clusters together based on their positions in the cluster tree. As a result, we ensured that each cluster would have at least 10 unique differentially expressed genes (DEGs). Differentially expressed genes in each cluster were identified using the FindAllMarker function within Seurat package and a corresponding p-value was given by the Wilcoxon's test followed by a Bonferroni correction.

GraphPad Prism software version 9.2.0 for Windows was used to perform multiple comparisons analysis among groups with a two-way ANOVA followed by Tukey's multiple comparisons test.

Cell type signature analysis
In order to annotate each cell type from the previous clustering, we referred to established studies and used signature gene sets for each cell type (Suppl Table 2). Treating the signature gene set for each cell type as a pseudogene, we evaluated the signature score for each cell in our dataset using the AddModuleScore function. Each cluster in our dataset was assigned with an annotation of its cell type by top signature scores within the cluster. To validate the identities of the tumor cell populations, we estimated copy number variants (CNV) via InferCNV (Version 1.4.0), using non-tumor and non-tumor-associated populations as reference. During the inferCNV run, genes expressed in fewer than five cells were filtered from the data set and the cut off was fixed at 0.1. Hidden Markov model (HMM) based CNV prediction was achieved and estimated CNV events were shown in a heatmap.
All tumor cells were subset and re-clustered using the analytical workflow described above. Eight clusters of tumor cells were obtained with distinctive transcriptomic profiles. By downsampling each cluster to 200 or fewer cells, we computed the correlation matrix between each tumor cell pairs and used the pheatmap R package (Version 1.0.12) (Kolde, 2019, https://CRAN.R-project.org/package=pheatmap) to make the correlation heatmap with unsupervised clustering. Gene signatures were tested on all eight tumor cell clusters using previously established hepatoblast, fibroblast, erythroid and neuroendocrine signature gene sets. A one-way ANOVA test was conducted for each signature score comparison across all eight tumor cell clusters and a corresponding p-value was computed.
In this analysis, we created customized gene set signatures for each cell population of interest. Using the DEGs obtained from FindAllMarker function, we included genes with log2 fold change > 2 and statistical significance (FDR q < 0.05) as the signature gene set.

Tumor-associated erythroid developmental analysis
Tumor-associated erythroid populations were extracted and integrated with the fetal liver erythroid or erythroblast populations from two publicly available datasets. We utilized the integration method based on commonly-expressed anchor genes by following the Seurat integration vignette to remove batch effects of samples sequenced with different technologies and possible artifacts so that the cells were comparable.
To evaluate the tumor-associated erythroid and tumor cell populations with respect to the fetal developmental stages, we first calculated a partition-based graph abstraction (PAGA) graph using SCANPY's (Version 1.4.2) sc.tl.paga() function and then used sc.tl.draw_graph() to generate the PAGA-initialized single-cell embedding of the cell types. The expression of markers was projected from the three fetal erythroid developmental markers (early, mid and late) to each tumor and erythroid cluster to generate a heatmap.

Pseudotime analysis
The erythroid population was exported as a Seurat object and then converted into a SingleCellExperiment sim object. Pseudotime analysis was conducted using the slingshot R package (Version 1.6.1). First, PCA decomposition was performed using the prcomp() function in the stats R package (Version 3.6.2). A diffusion map was then generated using the top layer annotation from the original Seurat object and the pseudotime trajectory was superimposed on the diffusion map. The starting point of the pseudotime trajectory was determined as the early erythroid population.

Cell-cell interaction analysis
We evaluated cell-cell interactions between two populations of interest using the CellPhoneDB package (Version 2.1.4). For each analysis, two input files were generated including a normalized gene expression matrix and a two-column metadata for cell names and annotations. The normalized gene expression matrix was obtained by using the NormalizeData function in Seurat with "RC" method specified. Statistical analysis of all available ligand-receptor pairs was performed on local computers.
To investigate the biologically relevant cell populations, we filtered the CellPhoneDB p-value.txt output file for ligand receptor pairs with the p-value less than 0.05, indicating statistically significant interactions, and generated customized columns and rows txt files. Dot plots were then plotted using these files to illustrate only the significant ligand-receptor interactions.

Image Processing
Stained sections were imaged with a Leica DM6B with 0.28 µm z-step size, using a 40× objective and LASX 3.7 software (Leica). Images were 3D-deconvoluted then an Extended Depth of Focus image was generated using LASX 3.7.

Data and custom code availability
Raw single-cell RNA sequencing FASTQ files and gene expression matrices files generated in this study have been deposited in the Gene Expression Omnibus (GEO) under the accession number GSE186975. Custom codes used in the manuscript are accessible via this github web link: https://github.com/angelussong/Hepatoblastoma_Analysis/

For manuscripts utilizing custom algorithms or software that are central to the research but not yet described in published literature, software must be made available to editors and reviewers. We strongly encourage code deposition in a community repository (e.g. GitHub). See the Nature Portfolio guidelines for submitting code & software for further information.

Data

Policy information about availability of data

All manuscripts must include a data availability statement. This statement should provide the following information, where applicable:

- Accession codes, unique identifiers, or web links for publicly available datasets
- A description of any restrictions on data availability
- For clinical datasets or third party data, please ensure that the statement adheres to our policy

Fetal liver erythroblasts and hepatocytes publicly available data used in this study are available at descartes.brotmanbaty.org (https://descartes.brotmanbaty.org/bbi/human-gene-expression-during-development/dataset/liver). The fetal liver erythroid publicly available data used in this study are available at ArrayExpress under the accession code E-MTAB-740714 (https://www.ebi.ac.uk/arrayexpress/experiments/E-MTAB-7407/).
Data and custom code availability
Raw single-cell RNA sequencing FASTQ files and gene expression matrices files generated in this study have been deposited in the Gene Expression Omnibus (GEO) under the accession number GSE186975 (https://www.ncbi.nlm.nih.gov/geo/query/acc.cgi?acc=GSE186975)
Expression matrices generated in this study were aligned against hg19 reference genome (GRCh37.p13) (https://www.ncbi.nlm.nih.gov/assembly/GCF_000001405.25/)
Custom codes used in the manuscript are accessible via this github web link: https://github.com/angelussong/Hepatoblastoma_Analysis/

Field-specific reporting

Please select the one below that is the best fit for your research. If you are not sure, read the appropriate sections before making your selection.

☒ Life sciences ☐ Behavioural & social sciences ☐ Ecological, evolutionary & environmental sciences

For a reference copy of the document with all sections, see nature.com/documents/nr-reporting-summary-flat.pdf

Life sciences study design

All studies must disclose on these points even when the disclosure is negative.

Sample size	Sample size calculations were not performed. The sample size was eventually decided based on the number of tumor cells captured which, based on previously published studies, were deemed sufficient for transcriptomic analysis. All patients treated at our institution with hepatoblastoma over a study period of 1 year were included.
Data exclusions	De-identified data were collected for this study. Data were not excluded.
Replication	Single-cell RNA sequencing of fresh tissues were performed once per patient because the samples came from surgical resection. For patient-derived spheroids (PDS) scRNAseq Analysis, PDS were analyzed twice along passages as described in methods in order to control stability of gene expression profile of PDS. PDS drug cytotoxicity tests and doubling time measurments were performed with one technical replicate and four or five biological replicates. all attempts at replication were successful for PDS scRNAseq and drug cytoxicity studies.<br>Processed single-cell RNA sequencing data needed for reproducibility of our analytical findings will be deposited in the NCBI GEO database to ensure replication of the analysis.
Randomization	Not applicable as this study does not include 2 or more treatment/intervention arms.
Blinding	Blinding was not relevant as there were not different treatment/interventions being studied.

Reporting for specific materials, systems and methods

We require information from authors about some types of materials, experimental systems and methods used in many studies. Here, indicate whether each material, system or method listed is relevant to your study. If you are not sure if a list item applies to your research, read the appropriate section before selecting a response.

Materials & experimental systems

n/a	Involved in the study	
☐	☒	Antibodies
☒	☐	Eukaryotic cell lines
☒	☐	Palaeontology and archaeology
☒	☐	Animals and other organisms
☐	☒	Human research participants
☒	☐	Clinical data
☒	☐	Dual use research of concern

Methods

n/a	Involved in the study	
☒	☐	ChIP-seq
☒	☐	Flow cytometry
☒	☐	MRI-based neuroimaging

Antibodies

Antibodies used	Rabbit anti-POSTN, Supplier, Invitrogen, Cat number: PA534641, dilution 1:200.<br>Rabbit anti-CHGA, Supplier: Abcam, Cat number: ab283265, Clone: RM1025, dilution 1:200.<br>Mouse anti-COL1, Supplier: Abcam, Cat number: ab6308, Clone COL-1, dilution 1:200.
Validation	CHGA and COL-1 primary antibodies were validated on tissues known to express the protein of interest (normal liver for COL1, Pancreas for CHGA).<br>CHGA antibody was validated by the manufacturer and sited in several studies: https://www.abcam.com/chromogranin-a-antibody-rm1025-ab283265.pdf<br>COL-1 antibody was validated by the manufacturer and sited in several studies:<br>https://www.abcam.com/collagen-i-antibody-col-1-ab6308.pdf<br>POSTN antibody was validated by the manufacturer: "Thermo Fisher Scientific is committed to adopting higher validation standards for the Invitrogen antibody portfolio. We have implemented additional specificity tests to help ensure the highest confidence levels in our products. You can identify the products that have already undergone this testing with the Advanced Verification badge, shown above. This badge can be found in antibody search results and at the top of product webpages. The data supporting the Advanced Verification status can be found in the product specific data galleries. To learn more about our testing standards, please visit Invitrogen Antibody Validation."

Human research participants

Policy information about studies involving human research participants

Population characteristics	Nine human hepatoblastoma surgical resection samples were collected for this study. Only de-identified information was collected for this study. The patient characteristics are summarized in Supplementary Data 1 of the manuscript.
Recruitment	Consecutive patients referred for hepatoblastoma resection were recruited for inclusion in to the study. No patient was excluded from the study along the collection time. All patient samples came from patients hospitatized at UCSF.
Ethics oversight	We included the following statement in the manuscript. The UCSF Institutional Review Board (IRB) committee approved the collection of these de-identified patient data included in this study. All relevant ethical regulations for work with human participants have been followed and informed consent was obtained from all participants.

Note that full information on the approval of the study protocol must also be provided in the manuscript.
