# Supplementary material for: Single-cell analysis of hepatoblastoma identifies tumor signatures that predict chemotherapy susceptibility using patient-specific tumor spheroids
Source: Nat Commun. 2022 Aug 25;13:4878. doi: 10.1038/s41467-022-32473-z (PMC9411569; doi:10.1038/s41467-022-32473-z)
Supplement: Supplementary file 1 — Supplementary Information [file 41467_2022_32473_MOESM1_ESM.pdf]

Supplementary Figure 1

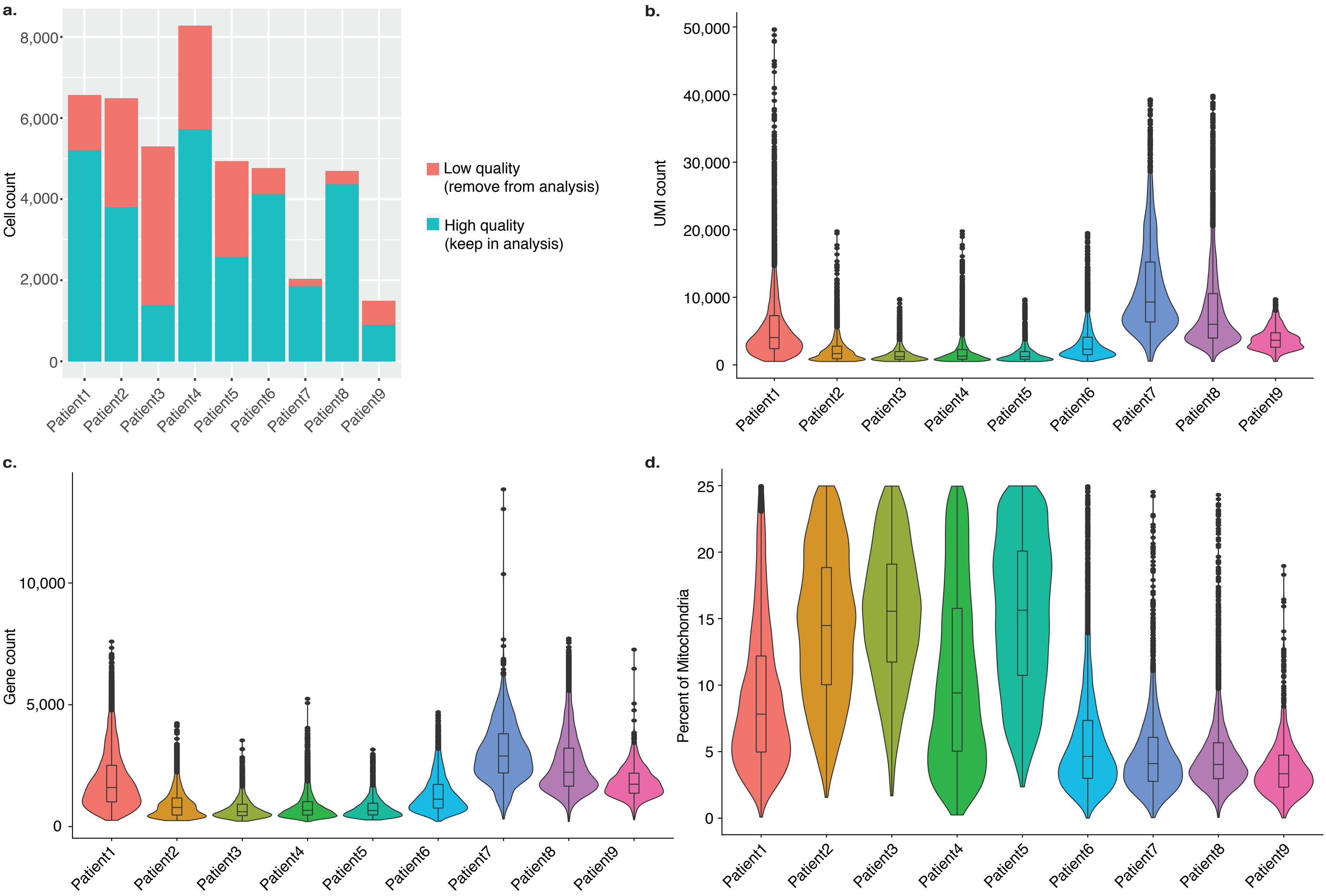

Supplementary Figure 1. Quality control of cells. a. Stacked bar charts of cells by overall quality for all patients. Low-quality cells are removed from downstream analysis. b. Violin and box plots of UMI counts per cell by patient (N = 29,968 cells, 9 patients). c. Violin and box plots of gene counts per cell by patient (N = 29,968 cells, 9 patients). d. Violin and box plots of percentage mitochondria per cell by patient (N = 29,968 cells, 9 patients). The box plots present the 25th percentile, the median, the 75th percentile, and outlying or extreme values. The whiskers of the box plots extend to a maximum of 1.5 times the size of the interquartile range. Source data are provided as a Source Data file.

Supplementary Figure 2

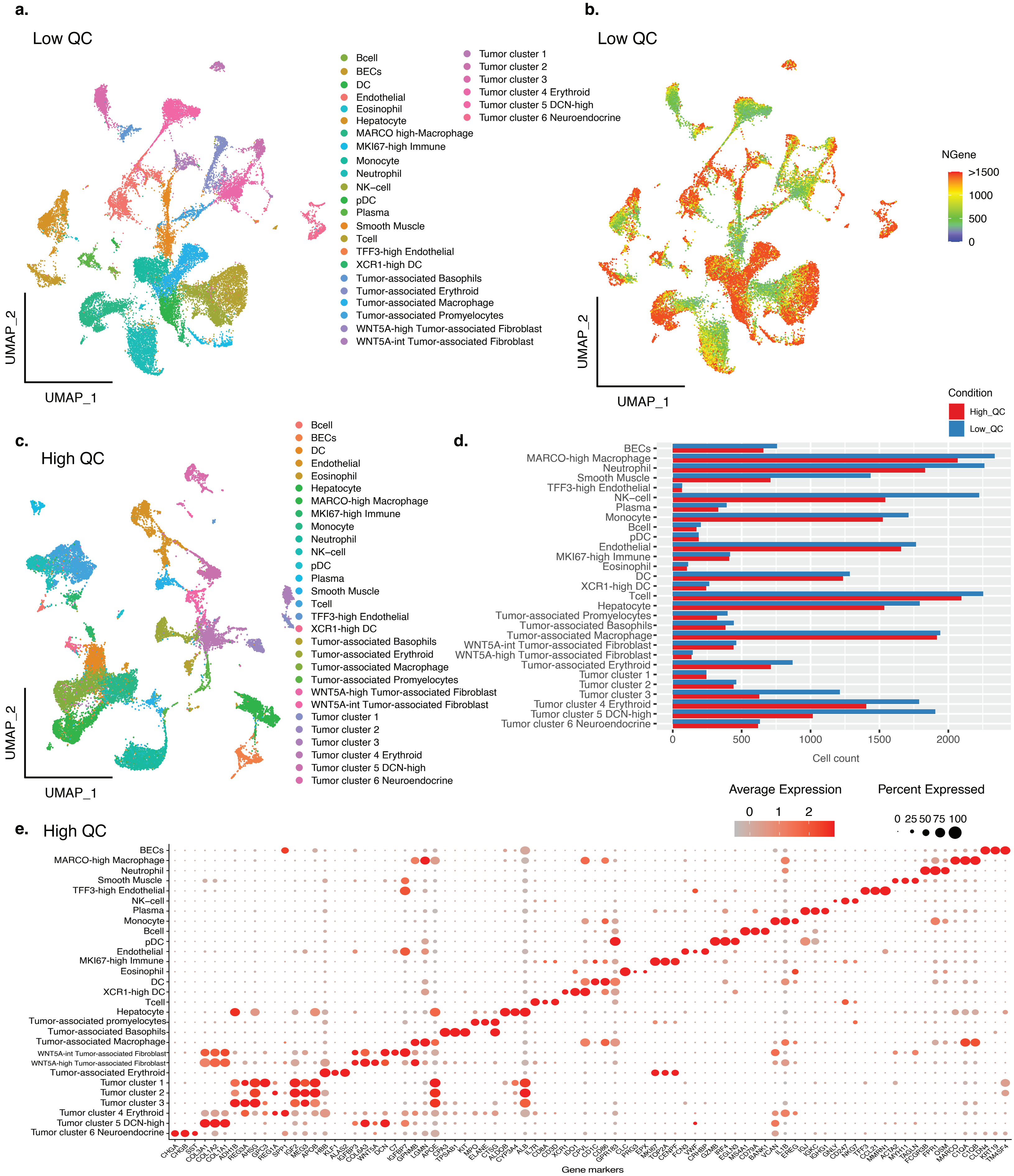

Supplementary Figure 2. Low QC and high QC comparison. a. UMAP of all cells passing the low QC threshold with final cell type annotation. b. UMAP heatmap of gene counts for all cells passing the low QC threshold with final cell type annotation. c. UMAP of all cells passing the high QC threshold with final cell type annotation. d. Bar chart of cell count comparison between low QC and high QC thresholds. e. Dot plot of all identified populations derived from the dataset with the high QC threshold, each characterized by three known cell type markers. Average expression was indicated by the color gradient and the percentage of marker expressed was represented by the dot size.

Supplementary Figure 3

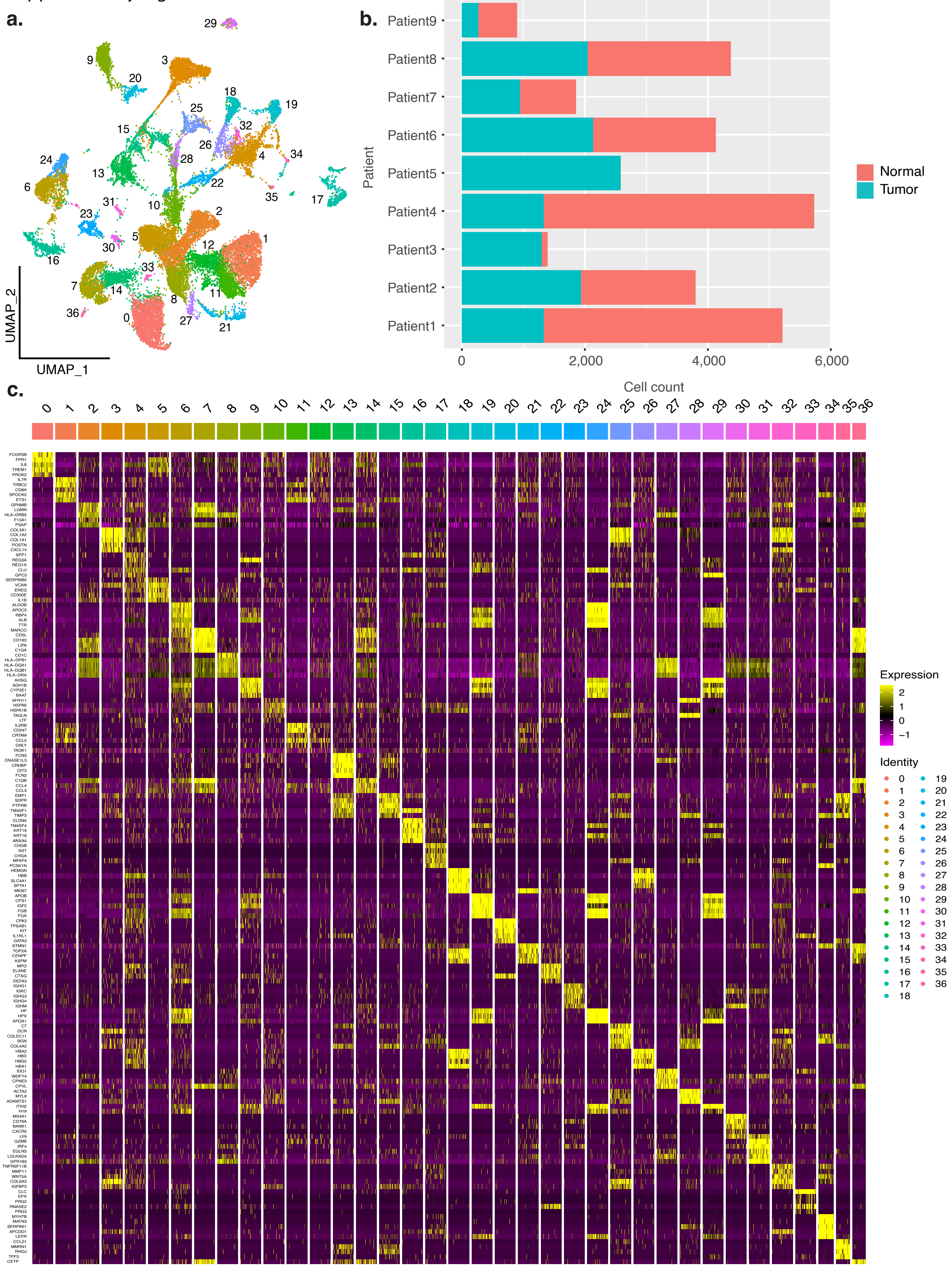

Supplementary Figure 3. Clustering of all cells passing QC from nine HBs. a. UMAP of all cells passing QC annotated by PCA-based clustering. b. Stacked bar chart of cell counts from tumor and normal samples for nine patients c. Heatmap of the top five most differentially expressed genes for each cluster. Scaled expression is shown in the color bar. Source data are provided as a Source Data file.

Supplementary Figure 4

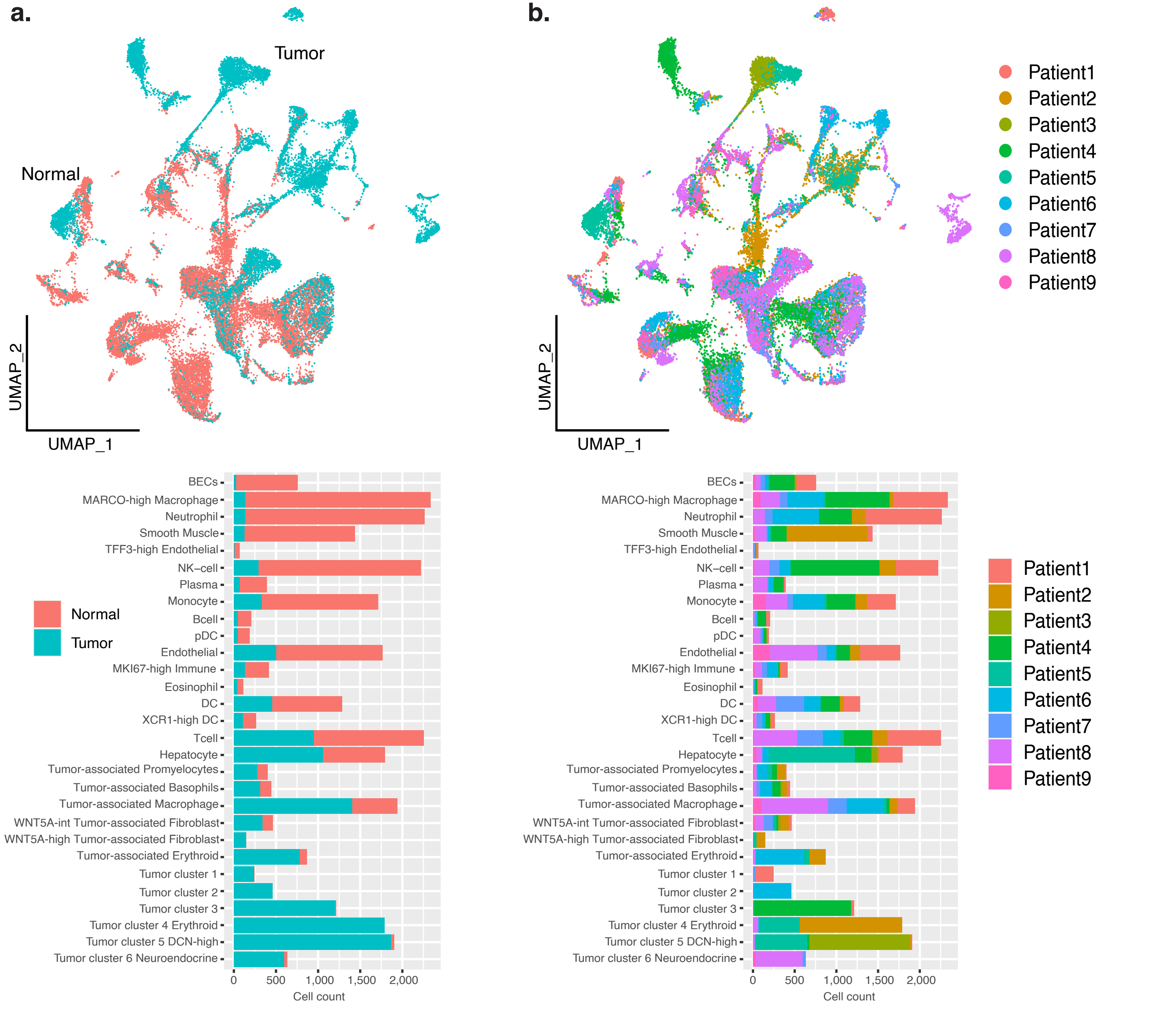

Supplementary Figure 4. Clustering by sample types and patients from nine HB patients. a. UMAP by sample types (tumor or adjacent normal samples) and stacked bar charts of each annotated clusters by sample types. b. UMAP by patients and stacked bar charts of each annotated cluster by patients. Source data are provided as a Source Data file.

Supplementary Figure 5

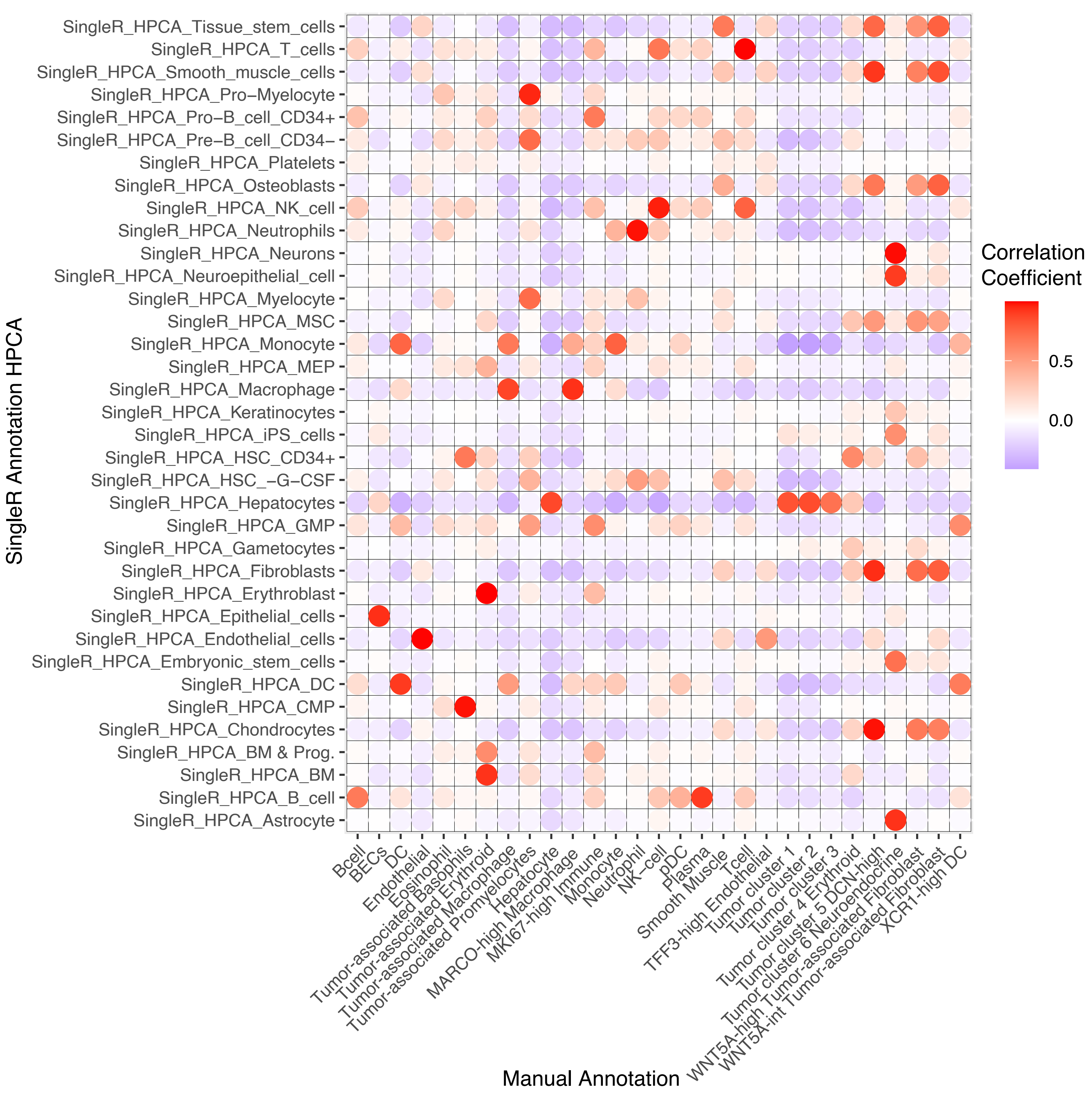

Supplementary Figure 5. Correlation heatmap of cell-type manual annotation and singleR automated annotation. Heatmap showing the correlation coefficient between manual annotation and singleR automated annotation. SingleR annotations are shown on the left and manual annotation at the bottom.

Supp Figure 6

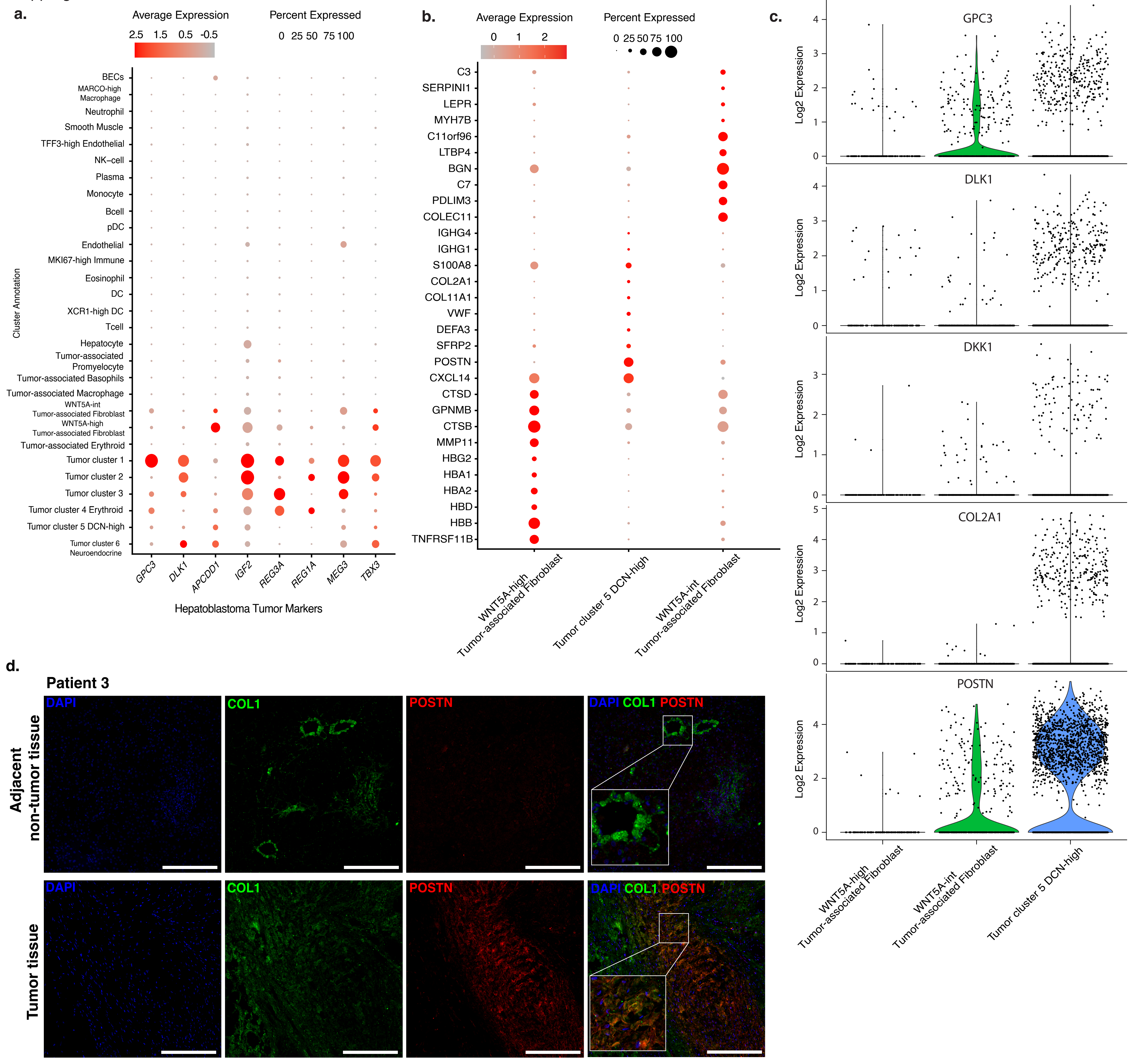

Supplementary Figure 6. Hepatoblastoma tumor gene expression pattern. a. Dot plot of 8 hepatoblastoma genes in all cell populations. b. Dot plot of the top 10 most differentially expressed genes within the DCN-high tumor cell cluster and two fibroblast clusters. Average expression levels are shown in the color bar and percent expression is indicated by the dot size. c. Violin plots of five representative hepatoblastoma tumor markers in the three clusters. d. Immunofluorescent staining for COL1 (green) and POSTN (red) of patient 3 adjacent non-tumor tissue and tumor tissue, N=2. Scale bar = 200 μm.

Supplementary Figure 7

a. Table of somatic mutations for CTNNB1 (exon 1 to exon 4)

Patient	Tissue / spheroids	CTNNB1 mutation ref: NP_001091679.1	CTNNB1 mutation ref: NM_001904.4
1	Normal	n.a	n.a
	Tumor	Exon 3-4 large deletion	
2	Normal	wt	wt
	Tumor	p.A5_A80del	c.14_241del
	PDS early	p.A5_A80del	c.14_241del
	PDS late	p.A5_A80del	c.14_241del
3	Normal	wt	wt
	Tumor	p.S45P	c.133T>C
4	Normal	wt	wt
	Tumor	p.S29_D32delinsY	c.86_94del
5	Normal	wt	wt
	Tumor	wt	wt
6	Normal	wt	wt
	Tumor	p.D32V	c.95A>T
	PDS early	p.D32V	c.95A>T
	PDS late	p.D32V	c.95A>T
7	Normal	wt	wt
	Tumor	p.S23_S33delinsP	c.67_97delinsT
	PDS early	p.S23_S33delinsP	c.67_97delinsT
	PDS late	p.S23_S33delinsP	c.67_97delinsT
8$	Normal	wt	wt
	Tumor*	p.A5_W66del	c.14_199del
	PDS early	p.A5_W66del	c.14_199del
	PDS late	p.A5_W66del	c.14_199del
9	Normal	wt	wt
	Tumor	wt	wt
	PDS early	wt	wt

* A third variant containing exon2 (normally spliced) but not exon 3 detected in tumor tissue only.

$ Splicing site moved from gene position 25,104 to 25,290 resulting to p.A5_W66del

b.

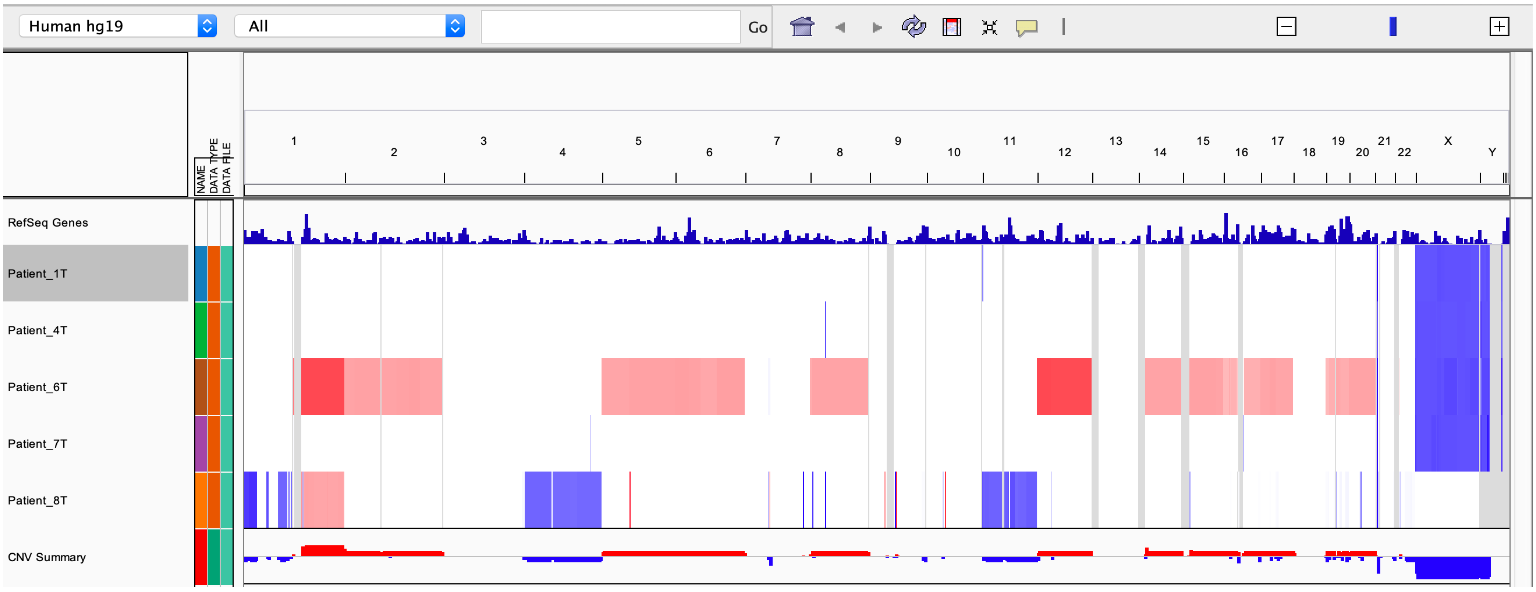

Supplementary Figure 7. CTNNB1 somatic mutation status and copy number alterations. a. CTNNB1 mutation status at the protein level (ref: NP_001091679.1) and at the mRNA level (ref: NM_001904.4) for the nine HB patients (tumor and adjacent non-tumor tissue) and for their corresponding PDS when applicable. b. Copy number alterations throughout the genome for five HB patients (patient 1, 4, 6, 7, and 8). Copy number changes are represented in color (red: amplification, blue: deletion).

Supplementary Figure 8

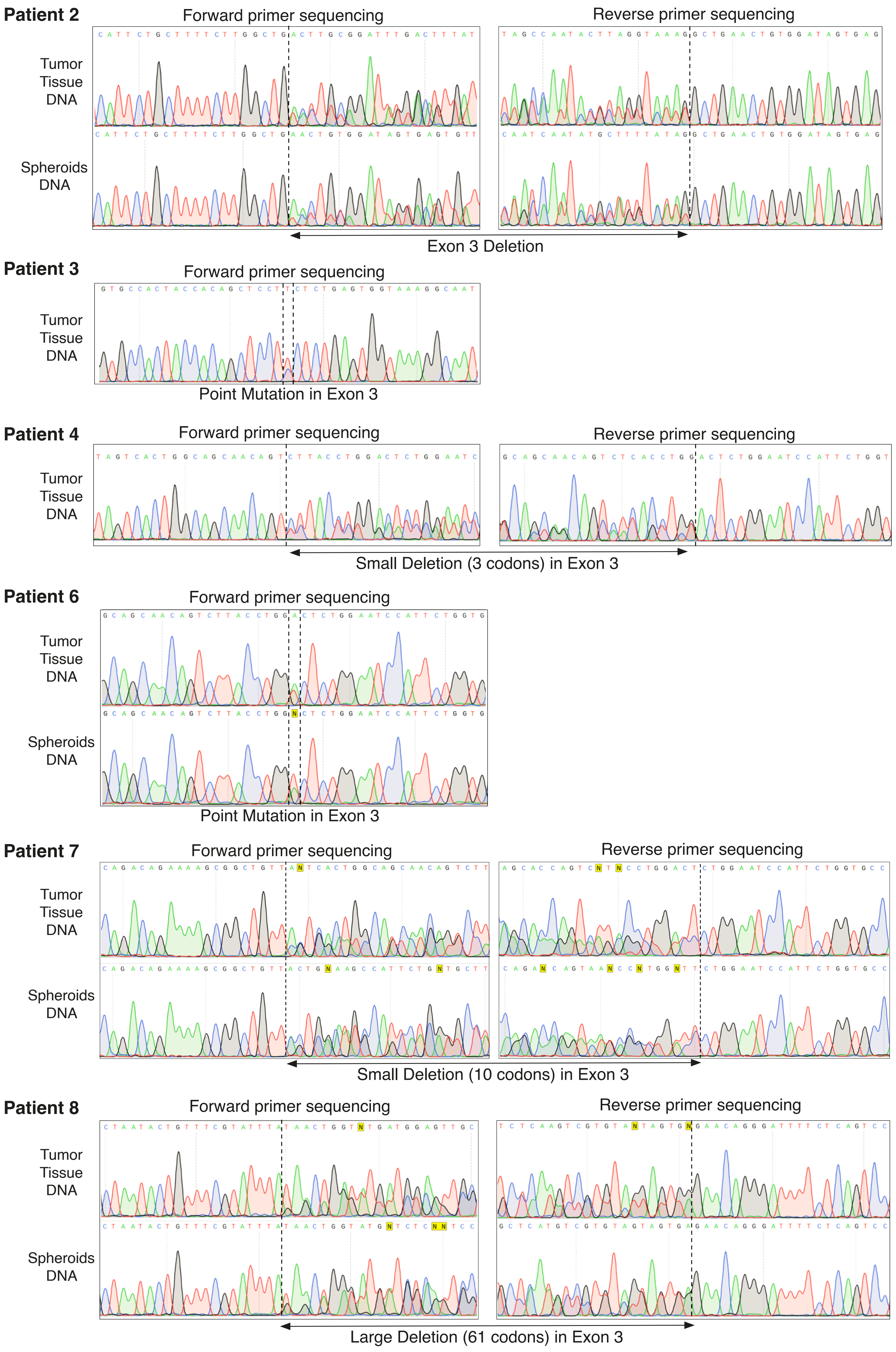

Supplementary Figure 8. Sanger traces of CTNNB1 mutation status analysis. Sanger traces (from forward and reverse primers when applicable) of mutation starting and stopping points (dotted lines) for CTNNB1 PCR products from DNA from patients 2, 3, 4, 6, 7 and 8 tumor tissue and from corresponding PDS when applicable.

Supplementary Figure 9

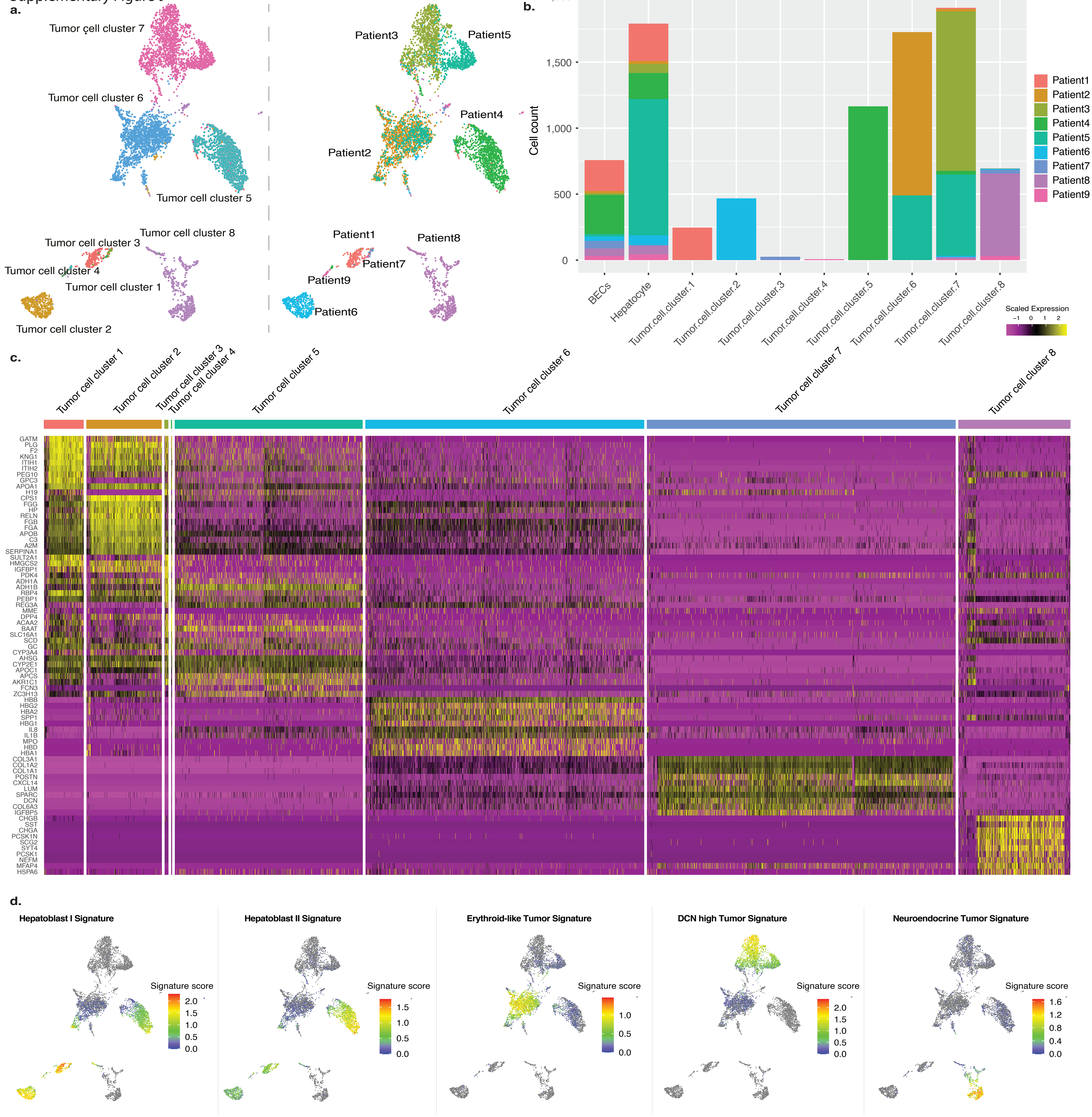

Supplementary Figure 9. Tumor cell analysis. a. UMAP of tumor cells by cluster (left) and patients (right). b. Stacked bar charts showing the cell counts for the eight tumor cell clusters and the two non-malignant epithelial cell types by patients. c. Heatmap of top 10 most differentially expressed genes of the eight tumor clusters. Scaled expression levels are shown in the color bar. d. Feature plots of the five tumor signature scores within the tumor cell UMAP. Computed signature scores are shown in the color bar. Source data are provided as a Source Data file.

Supplementary Figure 10

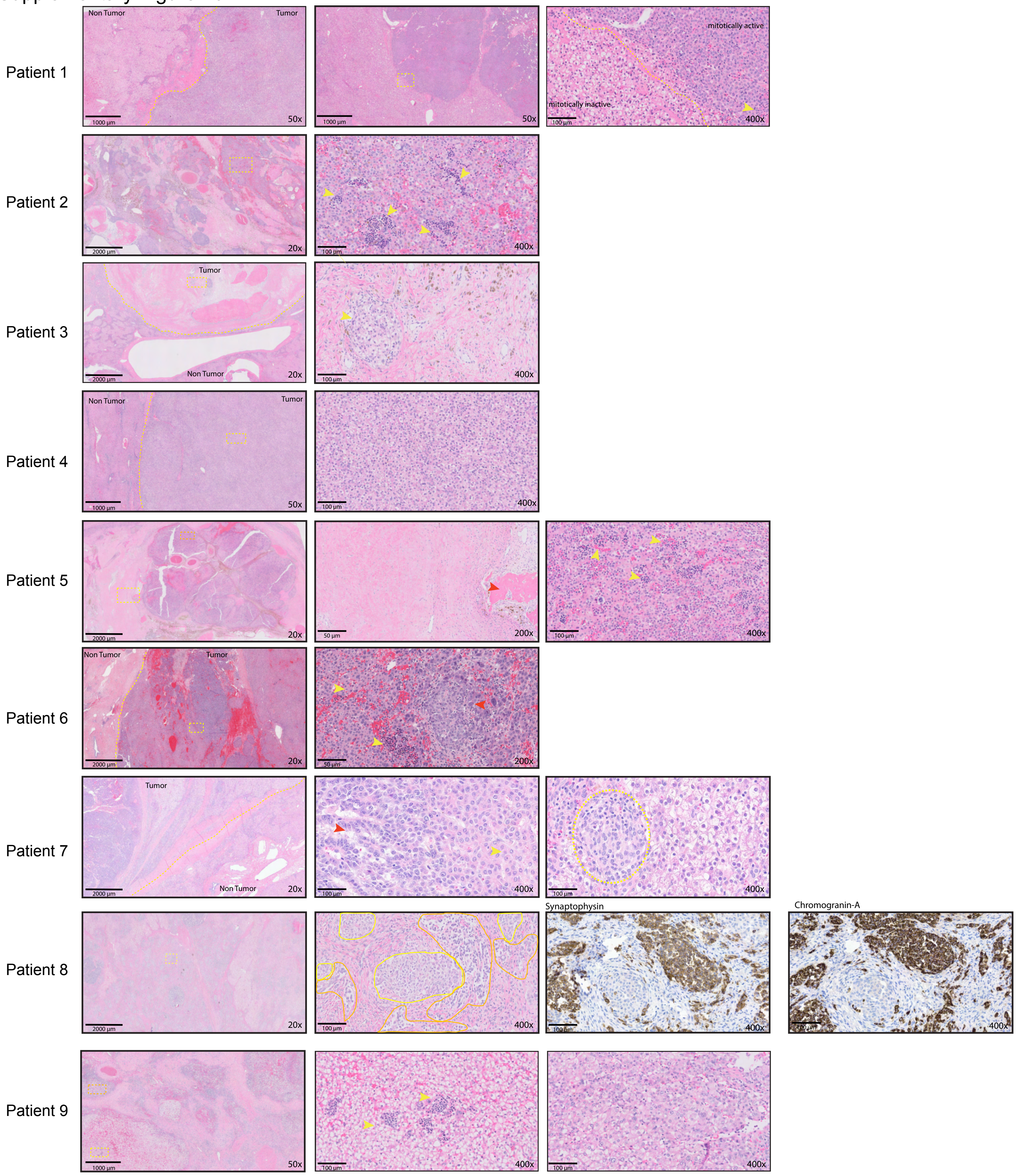

Supplementary Figure 10. Tumor tissue histology. H&E stainings showing representative histological features of tumor tissues for patients 1 to 9 and IHC stainings for Chromogranin-A and Synaptophysin for patient 8. Patient 1 had an epithelial-type tumor presenting a mitotically inactive (well differentiated) fetal pattern and a mitotic active (crowded) fetal pattern with scant extramedullary hematopoiesis (EMH) (arrow). 400x magnification image taken from yellow box area. Patient 2 had a mixed epithelial-mesenchymal-type tumor with 400x magnification (yellow box) demonstrating EMH (particularly erythropoiesis) (arrow) in a background of a fetal pattern. Patient 3 had a mixed epithelial-mesenchymal-type tumor, with 400x magnification (yellow box) presenting mostly fibrotic tissue with small islands of residual epithelial tumor (arrow). Patient 4 had a pure fetal epithelial-type tumor presenting a homogeneous well-differentiated/mitotically inactive fetal pattern demonstrated at 400x magnification (yellow box). Patient 5 had a mixed epithelial-mesenchymal-type tumor, with 200x magnification (yellow box, 2nd column) presenting large fibrotic area within tumor with residual osteoid-producing tumor (red arrow) as well as (orange box, third column) prominent EMH (yellow arrow) in a fetal pattern background. Patient 6 had an epithelial-type tumor, with 200x magnification (yellow box) presenting EMH (yellow arrow) in a fetal pattern background as well as a pleomorphic pattern (red arrow). Patient 7 had an epithelial-type tumor, with 400x magnification (second column) presenting a mix of embryonal pattern (red arrow) and fetal pattern (yellow arrow) as well as (third column) blastemal nests (circle) in a fetal pattern background. Patient 8 had a mixed epithelial-mesenchymal-type tumor, with 400x magnification (yellow box, second column) presenting fetal patterns (yellow encircled) with scattered and rounded nests with squamous differentiation (circle) and with cells with a higher nuclei/cytoplasm ratio at the periphery of nests of neuroendocrine pattern (orange encircled) positive for synaptophysin (third column) and chromogranin-A (fourth column). Patient 9 had a mixed epithelial-mesenchymal-type tumor, with 400x magnification (yellow box, second column) notably presenting EMH (yellow arrow) in a background of fetal and pleomorphic patterns and (orange box, third column) pleomorphic pattern.

Supplementary Figure 11

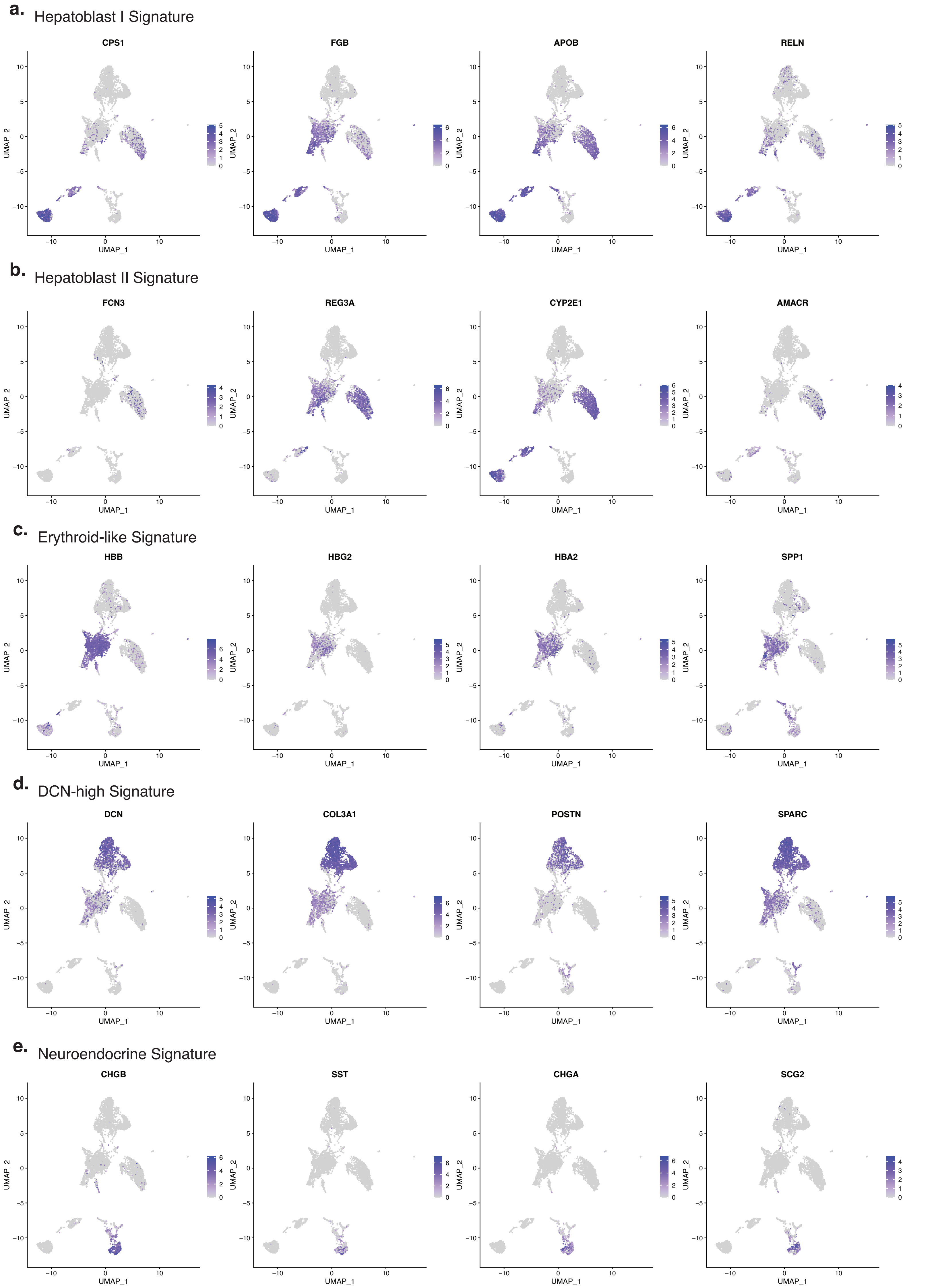

Supplementary Figure 11. Feature plots of representative markers for the five tumor subtype signatures. a. Feature plots of the top markers in Hepatoblast I signature including CPS1, FGB, APOB, and RELN. Expression levels are shown in color gradient. b. Feature plots of the top markers in Hepatoblast II signature including FCN3, REG3A, CYP2E1, and AMACR. Expression levels are shown in color gradient. c. Feature plots of the top markers in Erythroid-like signature including HBB, HBG2, HBA2, and SPP1. Expression levels are shown in color gradient. d. Feature plots of the top markers in DCN-high signature including DCN, COL3A1, POSTN, and SPARC. Expression levels are shown in color gradient. e. Feature plots of the top markers in Neuroendocrine signature including CHGB, SST, CHGA, and SCG2. Expression levels are shown in color gradient.

Supplementary Figure 12

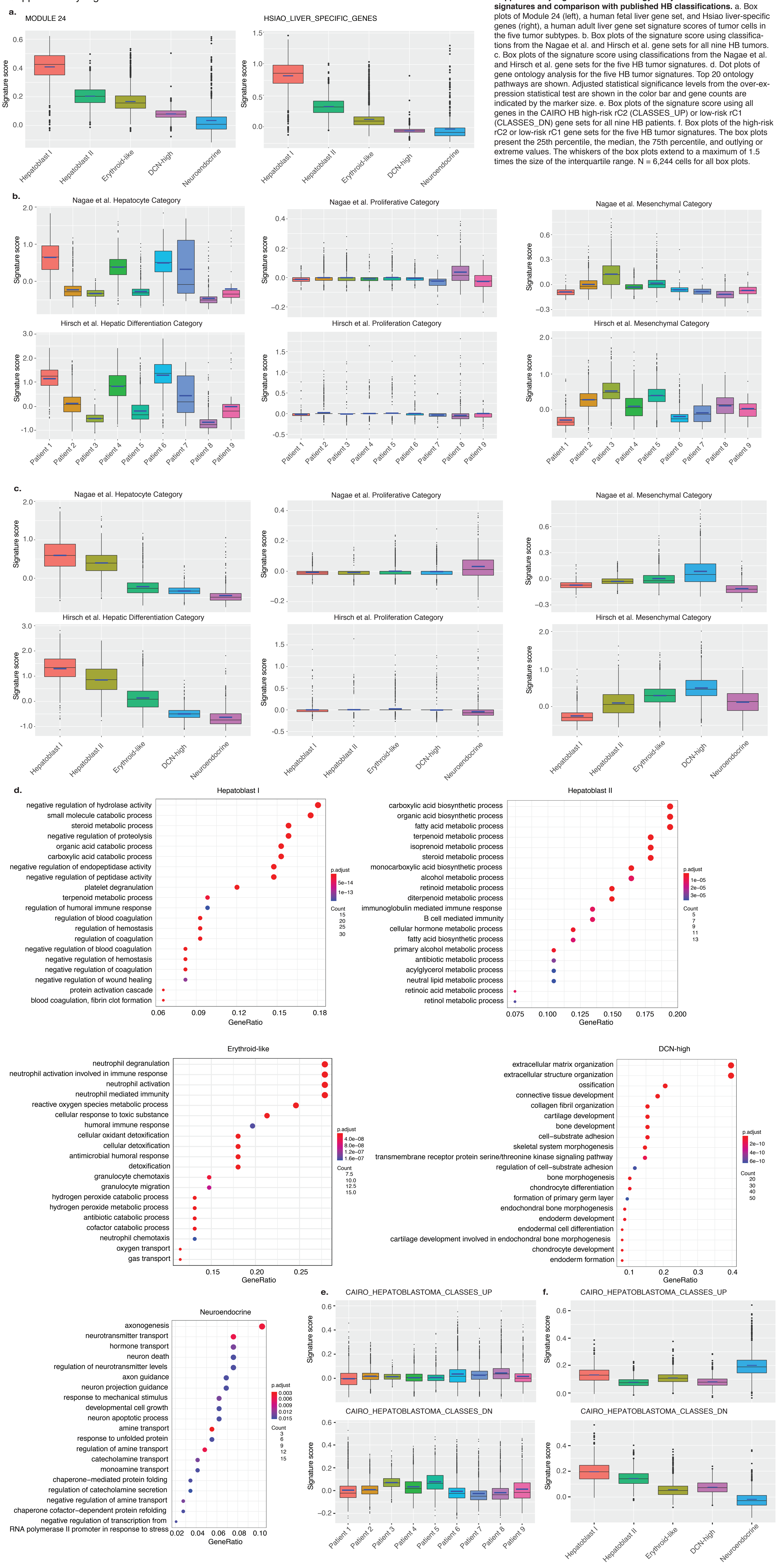

Supplementary Figure 12. Gene ontology analysis of the five HB tumor signatures and comparison with published HB classifications. a. Box plots of Module 24 (left), a human fetal liver gene set, and Hsiao liver-specific genes (right), a human adult liver gene set signature scores of tumor cells in the five tumor subtypes. b. Box plots of the signature score using classifications from the Nagae et al. and Hirsch et al. gene sets for all nine HB tumors. c. Box plots of the signature score using classifications from the Nagae et al. and Hirsch et al. gene sets for the five HB tumor signatures. d. Dot plots of gene ontology analysis for the five HB tumor signatures. Top 20 ontology pathways are shown. Adjusted statistical significance levels from the over-expression statistical test are shown in the color bar and gene counts are indicated by the marker size. e. Box plots of the signature score using all genes in the CAIRO HB high-risk rC2 (CLASSES_UP) or low-risk rC1 (CLASSES_DN) gene sets for all nine HB patients. f. Box plots of the high-risk rC2 or low-risk rC1 gene sets for the five HB tumor signatures. The box plots present the 25th percentile, the median, the 75th percentile, and outlying or extreme values. The whiskers of the box plots extend to a maximum of 1.5 times the size of the interquartile range. N = 6,244 cells for all box plots.

Supplementary Figure 13

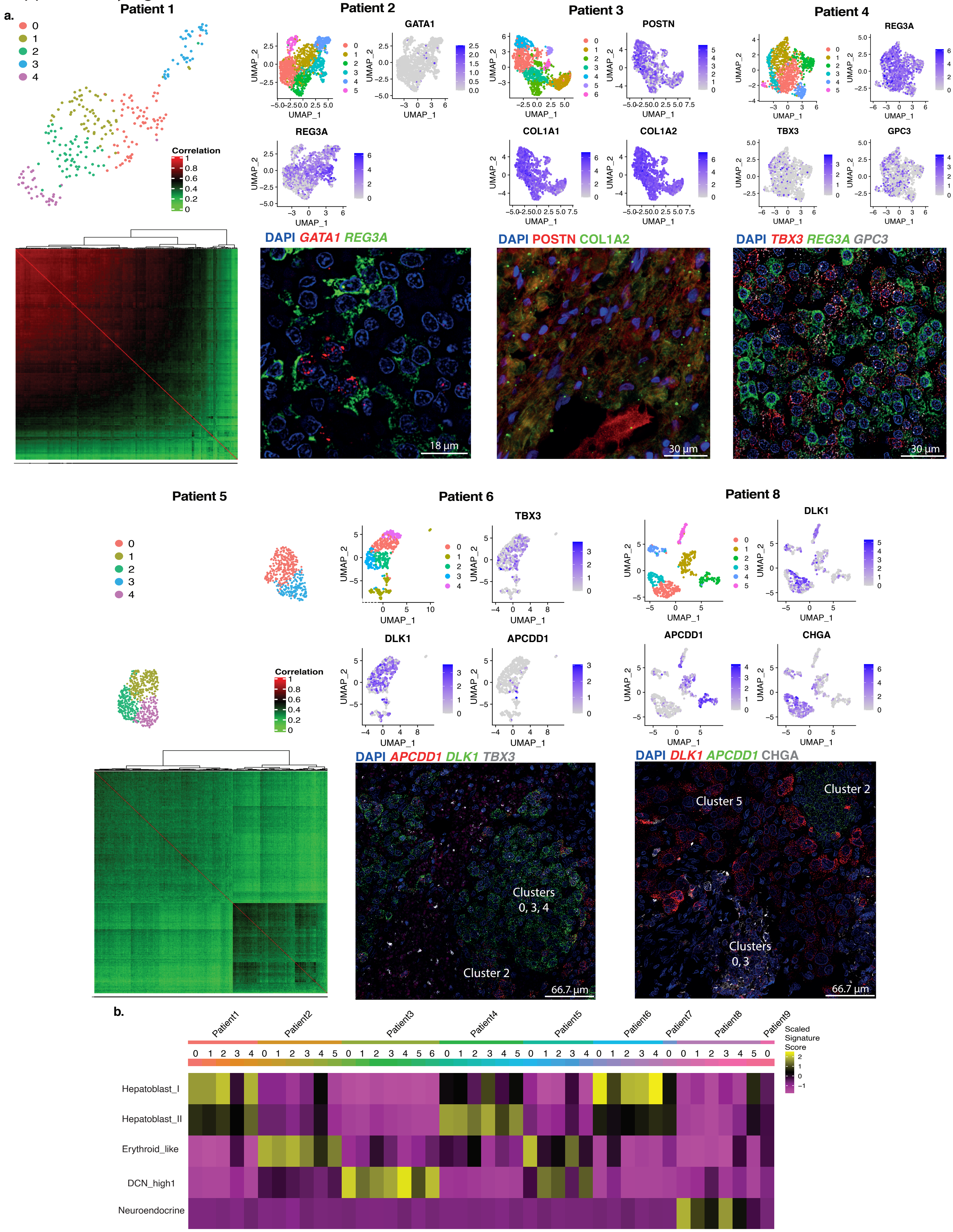

Supplementary Figure 13. Sub-clustering of tumor cells and staining within each HB patient. a. Sub-clustering of tumor cells in each patient. Tumor cell sub-clusters are shown in different colors. Correlation heatmaps are shown for patient 1 and patient 5 and correlation coefficients between each tumor cell pair are shown in the color bar. Representative markers for patients 2, 3, 4, 6, 8 are shown in feature plots, and FISH and immunofluorescent stains show the expression of those tumor markers for patient 2 (*GATA1* - red, *REG3A* - green), patient 3 (POSTN - red, COL1 - green), patient 4 (*REG3A* - green, *TBX3* - red, *GPC3* - white), patient 6 (*APCDD1* - red, *DLK1* - green, *TBX3* - white) and patient 8 (*DLK1* - red, *APCDD1* - green and CHGA - white). b. Heatmap of the five tumor subtype signature scores in each sub-clusters from the nine HB patients. Scaled signature scores are shown in the color bar.

Supplementary Figure 14

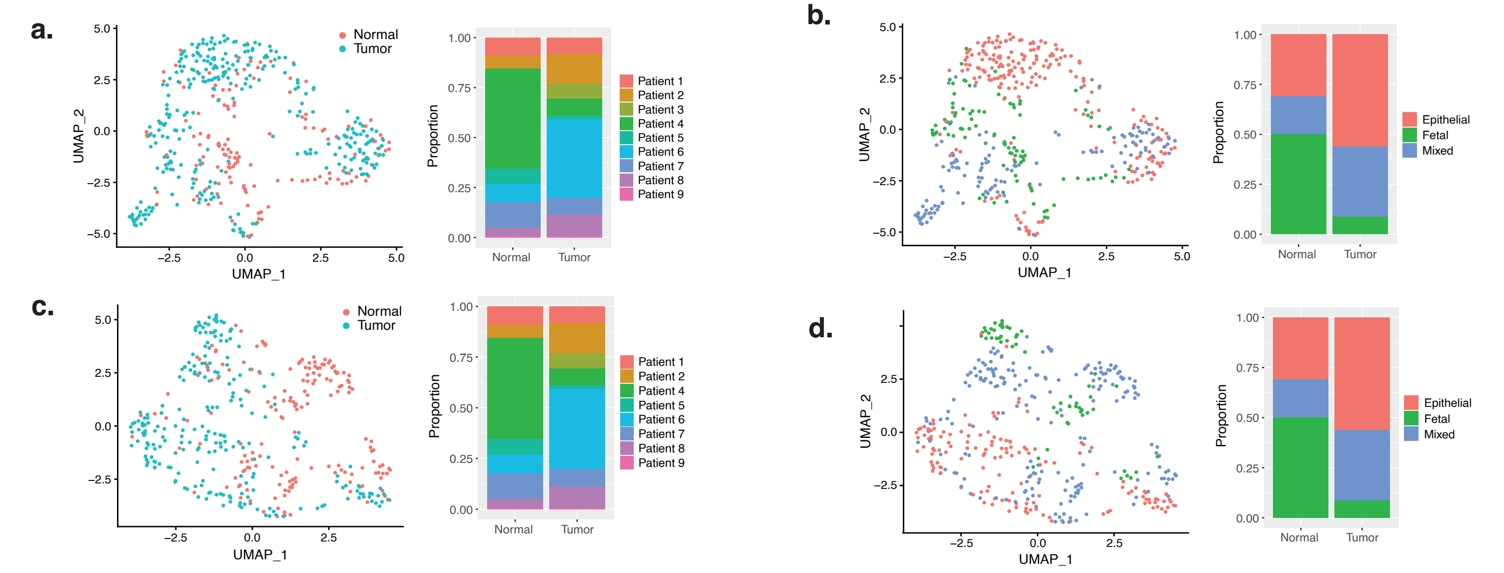

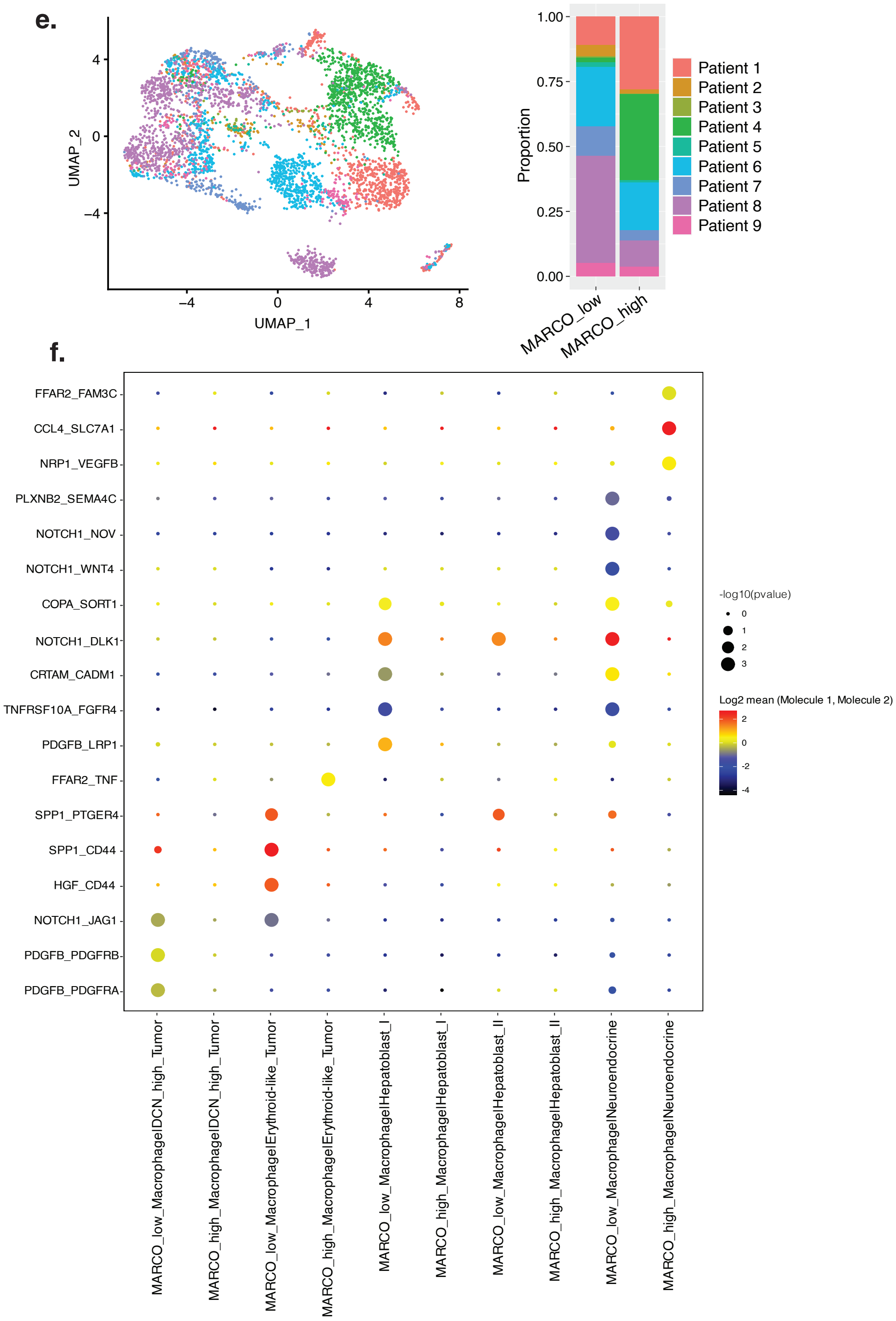

Supplementary Figure 14. Analysis of basophils and pro-myelocytes from HB tumors and normal liver, and ligand-receptor interactions between HB TAMs and cells expressing each of the five tumor signatures. a. UMAP of all basophils from all nine patients annotated by sample type (left) and bar graph showing the proportional contribution of basophils from each patient to each sample type (right). b. UMAP of all basophils from all nine patients annotated by histological subtype (left) and bar graph showing the proportional contribution of basophils from each histological subtype to each sample type (right). c. UMAP of all pro-myelocytes from all nine patients annotated by sample type (left) and bar graph showing the proportional contribution of pro-myelocytes from each patient to each sample type (right) d. UMAP of all pro-myelocytes from all nine patients annotated by histological subtype (left) and bar graph showing the proportional contribution of pro-myelocytes from each histological subtype to each sample type (right). e. UMAP of all macrophages from all 9 patients annotated by patient(left) and the proportional contribution from each patient to the MARCOLow and MARCOHi macrophage clusters. f. Dot plot of significant ligand-receptor interactions between MARCOLow macrophages and tumor cells expressing each HB signature. Mean expressions of ligand and receptor pairs are shown in the color bar. Statistical significance levels (random permutation test) are indicated by the marker size.

Supplementary Figure 15

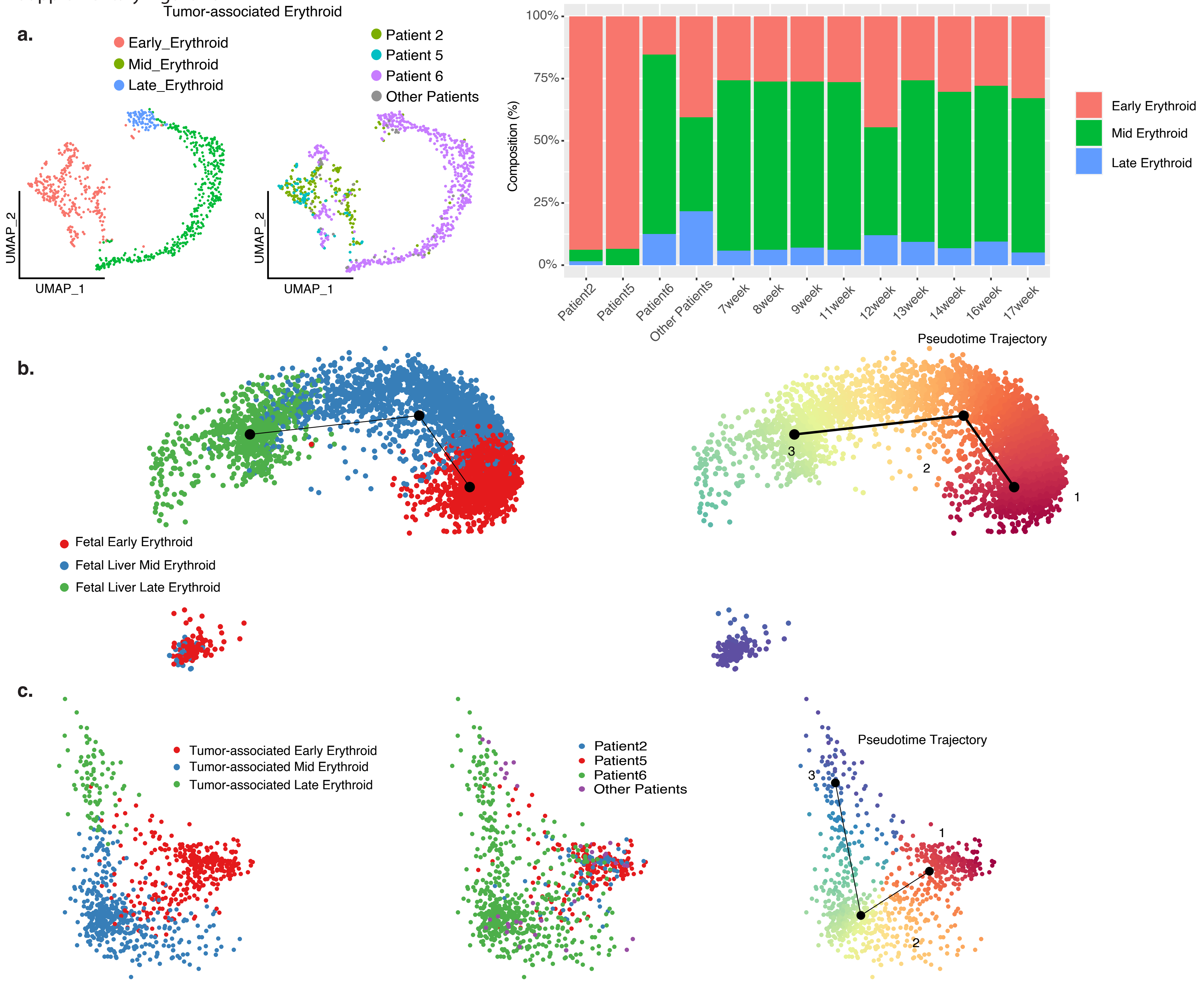

Supplementary Figure 15. Pseudotime analysis of tumor-associated erythroid cells. a. Left panel, UMAP of HB-associated erythroid cells annotated by the three developmental stages and patients. Right panel, stacked bar charts of HB-associated erythroid cells in patients 2, 5 and 6 compared with the fetal liver dataset from Popescu. et al. b. Clustering and pseudotime trajectory of fetal liver erythroid by the three developmental stages. c. Pseudotime trajectory of HB-associated erythroid cells by the three developmental stages and by patient. Source data are provided as a Source Data file.

Supplementary Figure 16

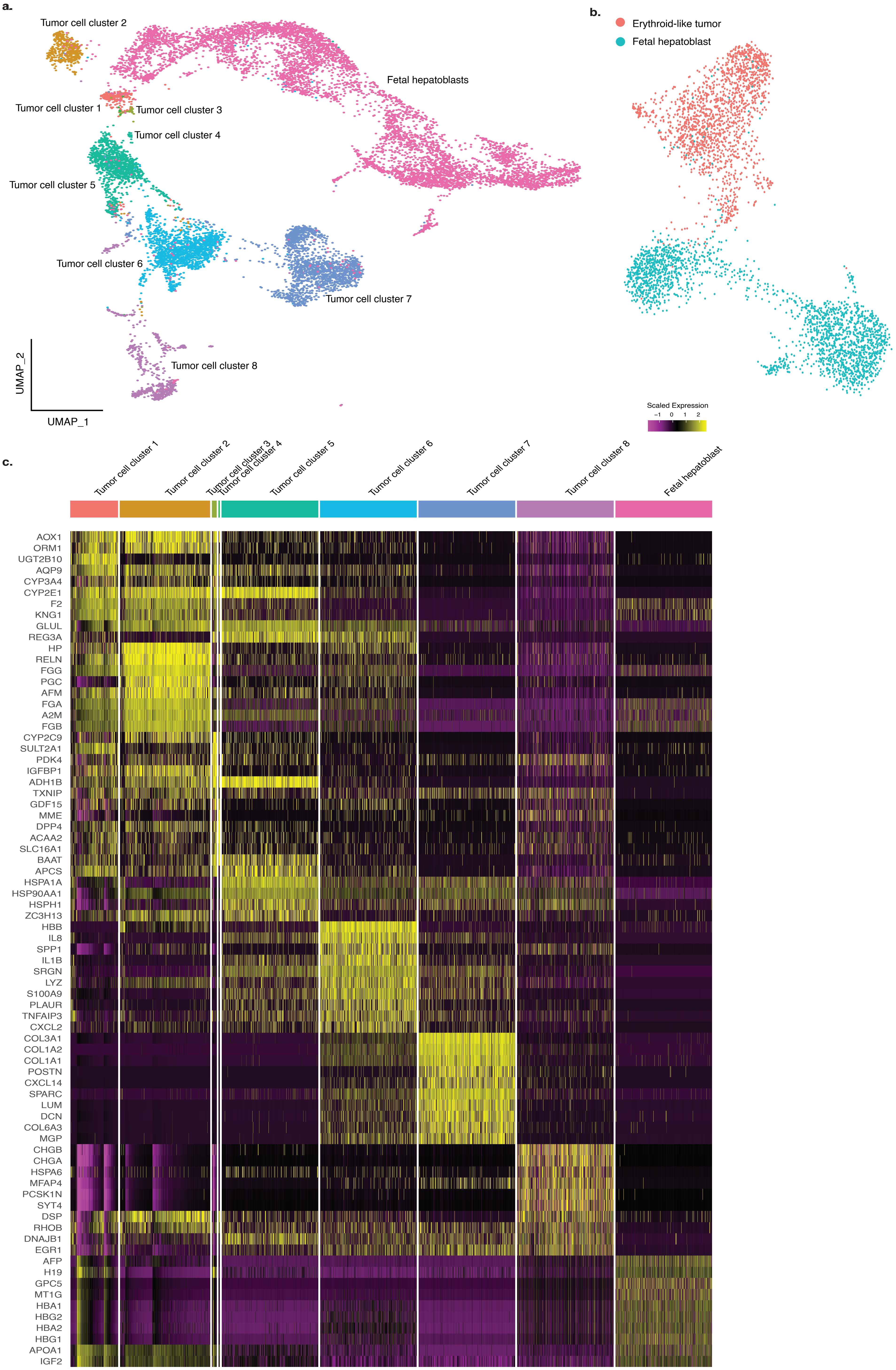

Supplementary Figure 16. Integrated analysis of tumor cells and fetal liver hepatoblasts. a. Integrated UMAP of tumor cells with fetal liver hepatoblasts. b. Integrated UMAP of Erythroid-like tumor cells with fetal liver hepatoblasts. c. Heatmap of top 10 most differentially expressed genes in each tumor cell cluster and reference fetal

Supplementary Figure 17

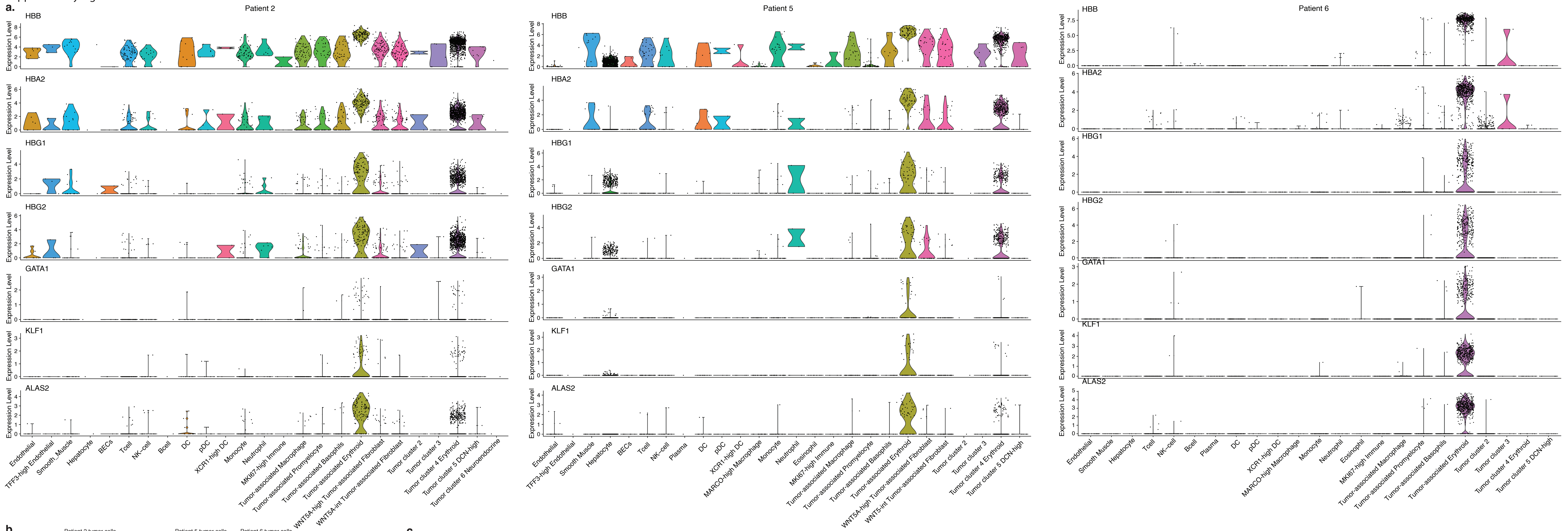

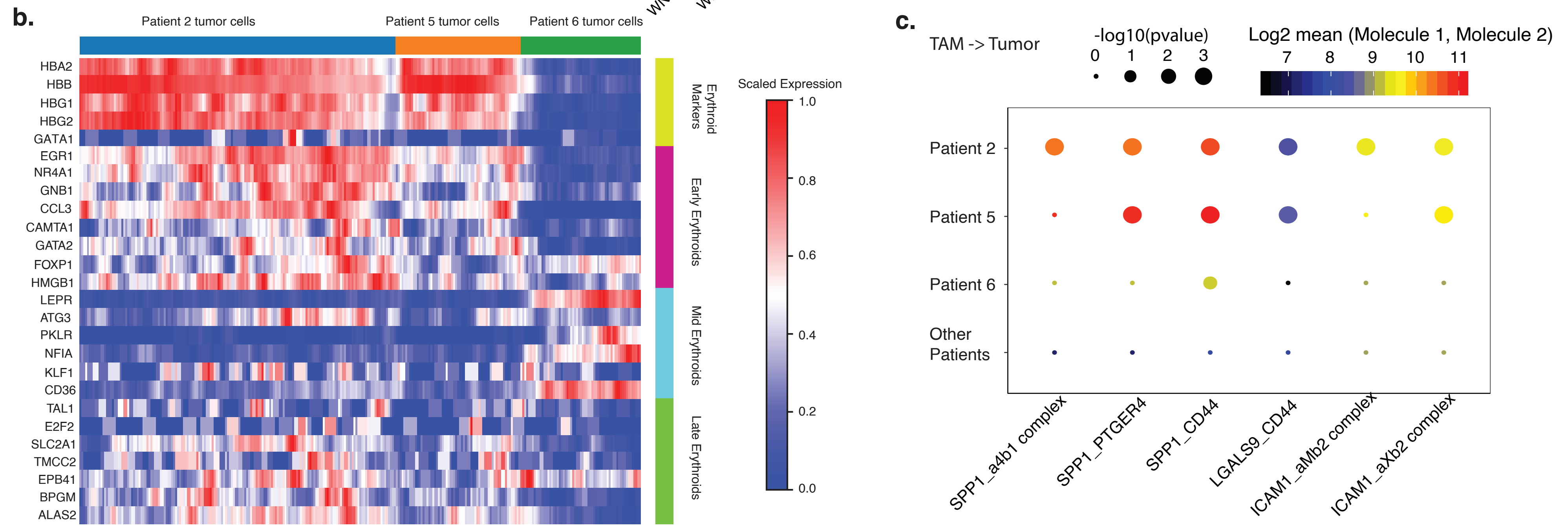

Supplementary Figure 17. Erythroid marker expressions in tumor cells of HB patients. a. Violin plots of seven canonical erythroid markers in all cell types in patients 2, 5 and 6 after ambient RNA decontamination. b. Supervised heatmap showing the expression of canonical erythroid markers and representative markers of three erythroid developmental stages in patients 2, 5 and 6 tumor cells. c. Representative ligand-receptor interactions between TAMs and tumor cells for patients 2, 5, 6 and other patients.Mean expressions of ligand and receptor pairs are shown in the color bar. Statistical significance levels (random permutation test) are indicated by the marker size.

Supplementary Figure 18

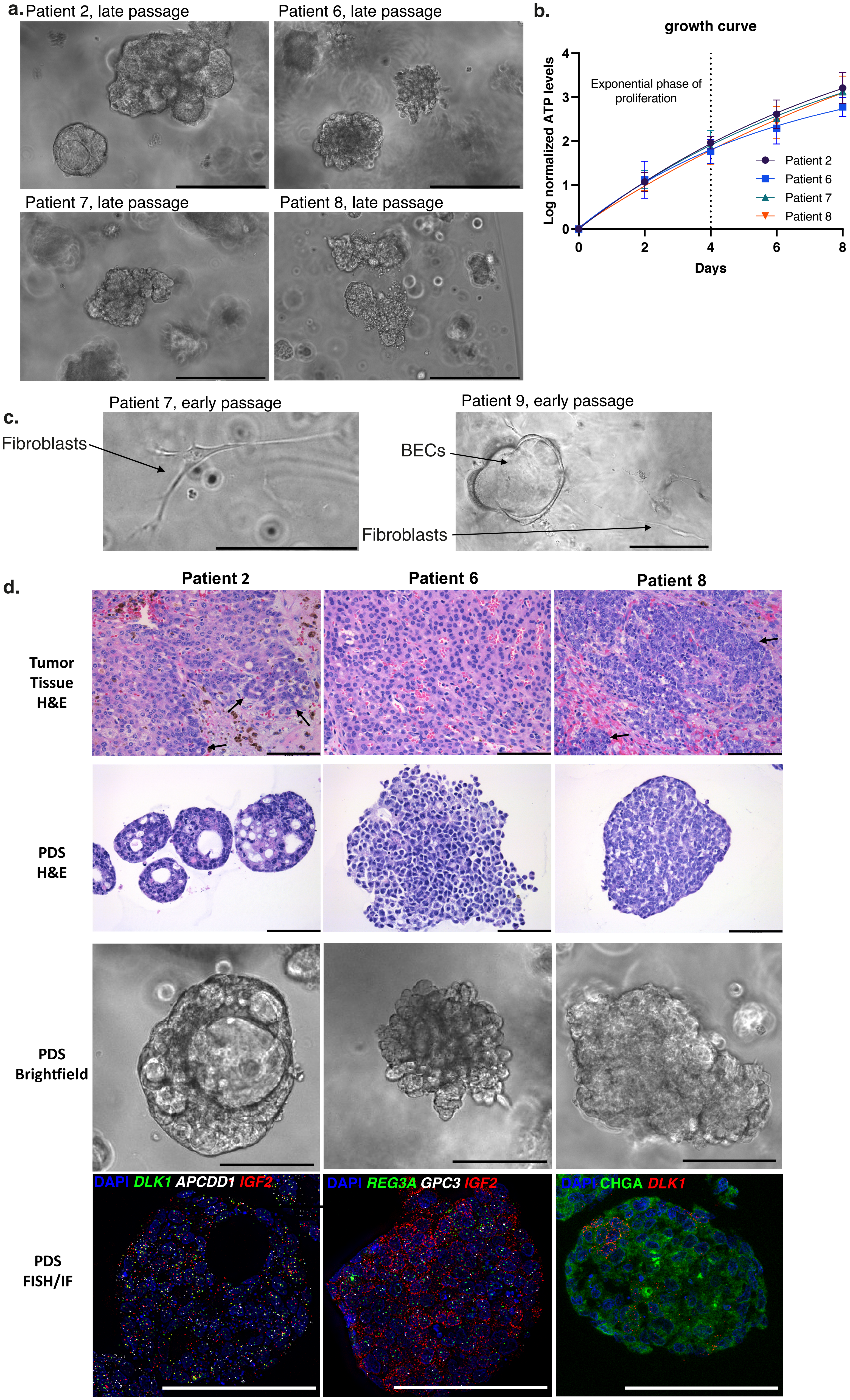

Supplementary Figure 18. Patient-derived-spheroid characteristics and histologic comparison with tumor tissue. a. Brightfield images of patients 2, 6, 7 and 8 tumor spheroids at late passages. Scale bar = 200 µm. b. PDS growth curves measured by ATP quantification every other day during 8 days after seeding. N = 5 for PDS from patient 2, 7 and 8 and N = 4 for patient 6. Data present mean +/- SD. c. Brightfield images of nearby population of patients 7 and 9 cultivated cells, identified as fibroblast-like cells and BEC-like cells. Scale bar = 100 µm. d. H&E stains of tumor tissues and PDS, and brightfield images of PDS from patients 2, 6 and 8. Arrows show cholangioblastic features and cells with a higher nuclei/cytoplasm ratio in patient 2 and 8 tumor tissues, respectively. For Patient 2 PDS, FISH staining show DLK1 (green), APCDD1 (white) and IGF2 (red). For Patient 6 PDS, FISH staining show REG3A (green), GPC3 (white) and IGF2 (red), N=2. For Patient 8 PDS, FISH/Immunofluorescence staining show CHGA (green) and DLK1 (red). Scale bar = 100 µm. Source data for growth curve are provided as a Source Data file. Early passage correspond to passage 2 for patients 2, 8, 9 and passage 3 for patients 6 and 7. Late passage correspond to passage 10 for patients 2, 6 and 8, and passage 11 for patient 7.

Supplementary Figure 19

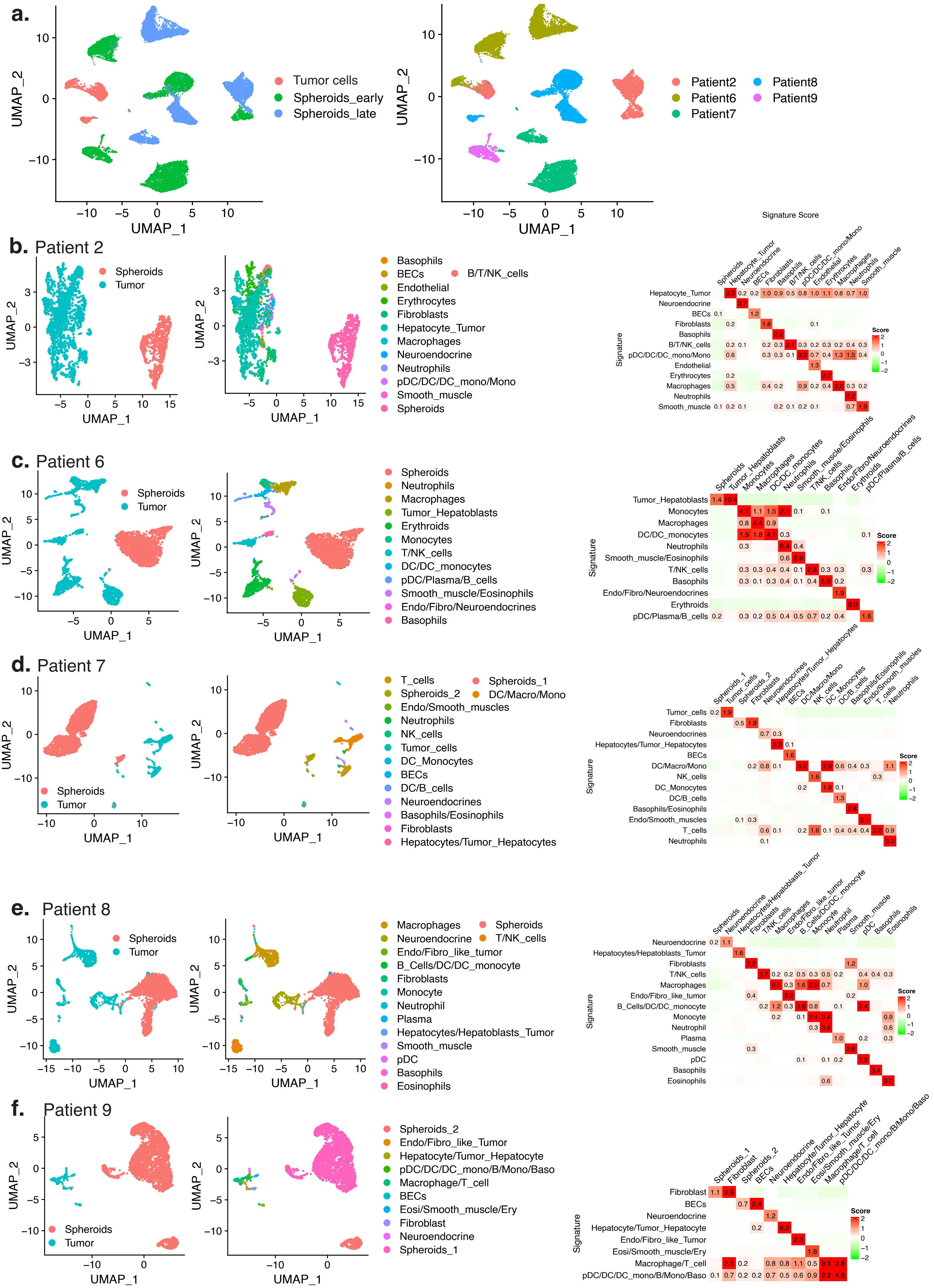

Supplementary Figure 19. Broad comparison of freshly isolated tumor cells and patient-derived-spheroids, and identification of spheroid parent cells. a. UMAP projection of merged scRNA-seq data of cells from tumor tissue and PDS at early and late passage for patients 2, 6, 7, 8 and 9. b.-f. (left panel) UMAP projection colored by origin (spheroids or tumor) or by cluster annotation (generated from initial annotation) (Figure 1). b.-f. (right panel) Heatmap illustrating signature scores of every cluster generated from signatures of cells from tumor tissue clusters (b. patient 2, c. patient 6, d. patient 7, e. patient 8, f. patient 9). Early passage correspond to passage 2 for patients 2, 8, 9 and passage 3 for patients 6 and 7. Late passage correspond to passage 10 for patients 2, 6 and 8, and passage 11 for patient 7.

Supplementary Figure 20

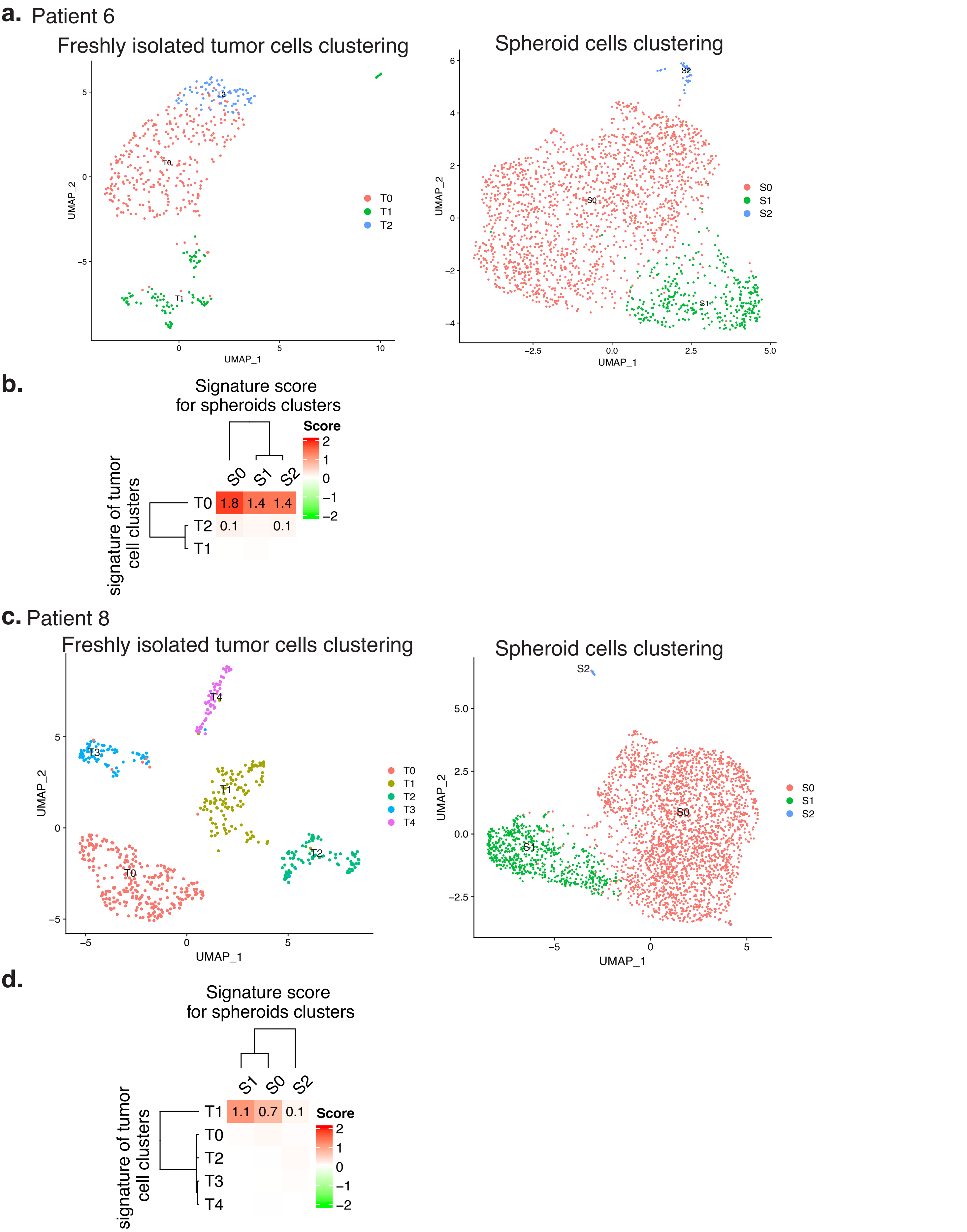

Supplementary Figure 20. Identification of a specific subpopulation of patient-derived-spheroid parent cells. a., c. UMAP projection of freshly isolated tumor cells and spheroids at early passage colored by subclusters. b., d. Signature score of PDS subclusters calculated with freshly isolated tumor cell signatures. (a., b. patient 6 and c., d. patient 8).

Supplementary Figure 21

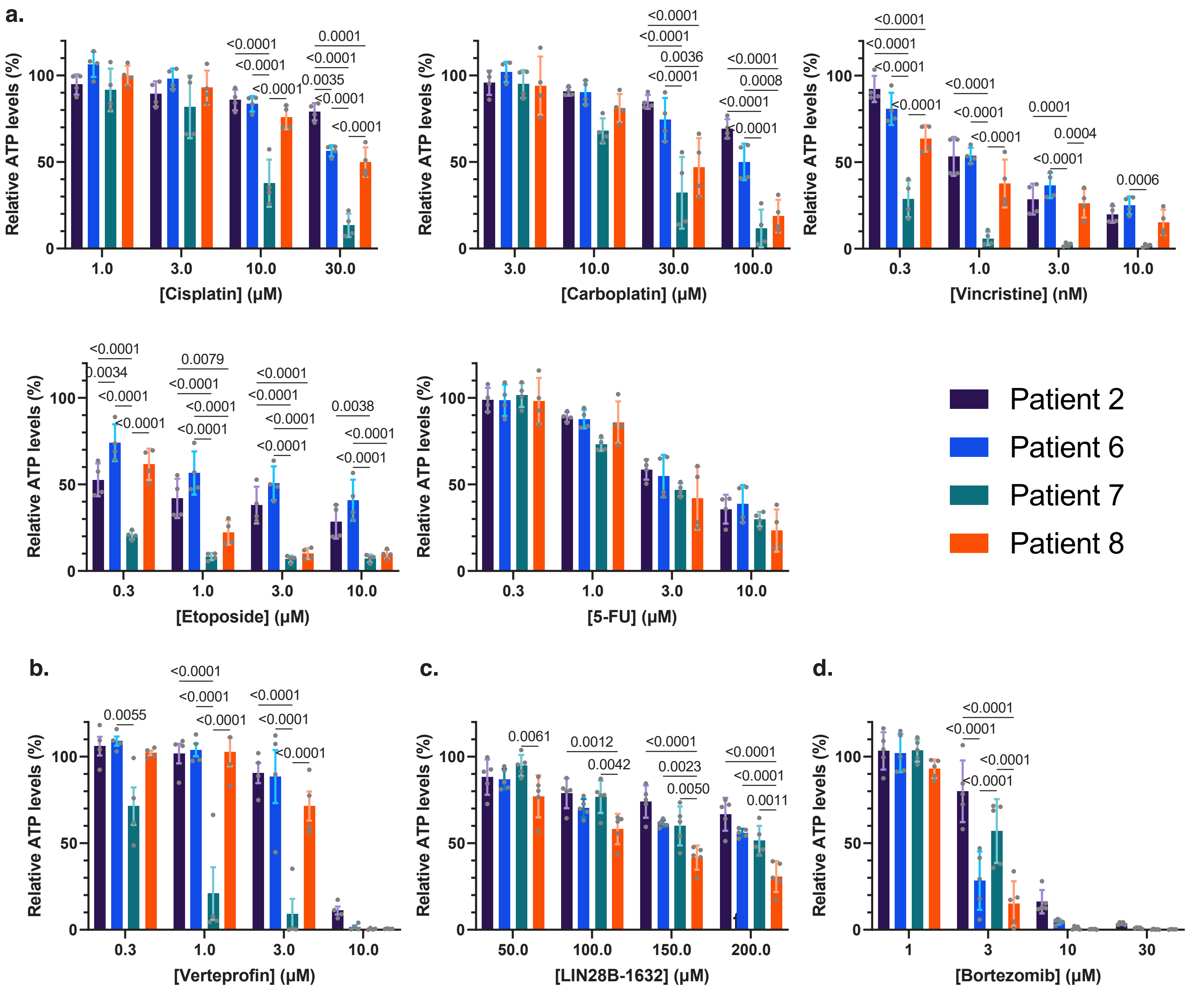

Supplementary Figure 21. Pharmacologic testing of patient-derived HB spheroids reveals patient-specific treatment responses. a. Cell viability measured by ATP quantification after a 4-day treatment with five chemotherapy drugs (cisplatin, carboplatin, etoposide, vincristine and 5-fluorouracil [5-FU]) b. Cell viability measured by ATP quantification after a 4-day treatment with the YAP1 inhibitor verteporfin. c. Cell viability measured by ATP quantification after a 4-day treatment with the LIN28 pathway inhibitor LIN28B-1632. d. Cell viability measured by ATP quantification after a 4-day treatment with the proteasome inhibitor bortezomib. Drug cytotoxicity data are presented as mean +/- SEM, N=4 for cisplatin, Carboplatin, vincristine, etoposide, 5FU and verteporfin, N=5 for LIN28B-1632, Bortezomib, Two-way ANOVA was performed, followed by Tukey's multiple comparisons test. p-values are indicated on graphs (p<0.0001 is indicated when p-value is below this threshold).Source data are provided as a Source Data file.

Supplementary Figure 22

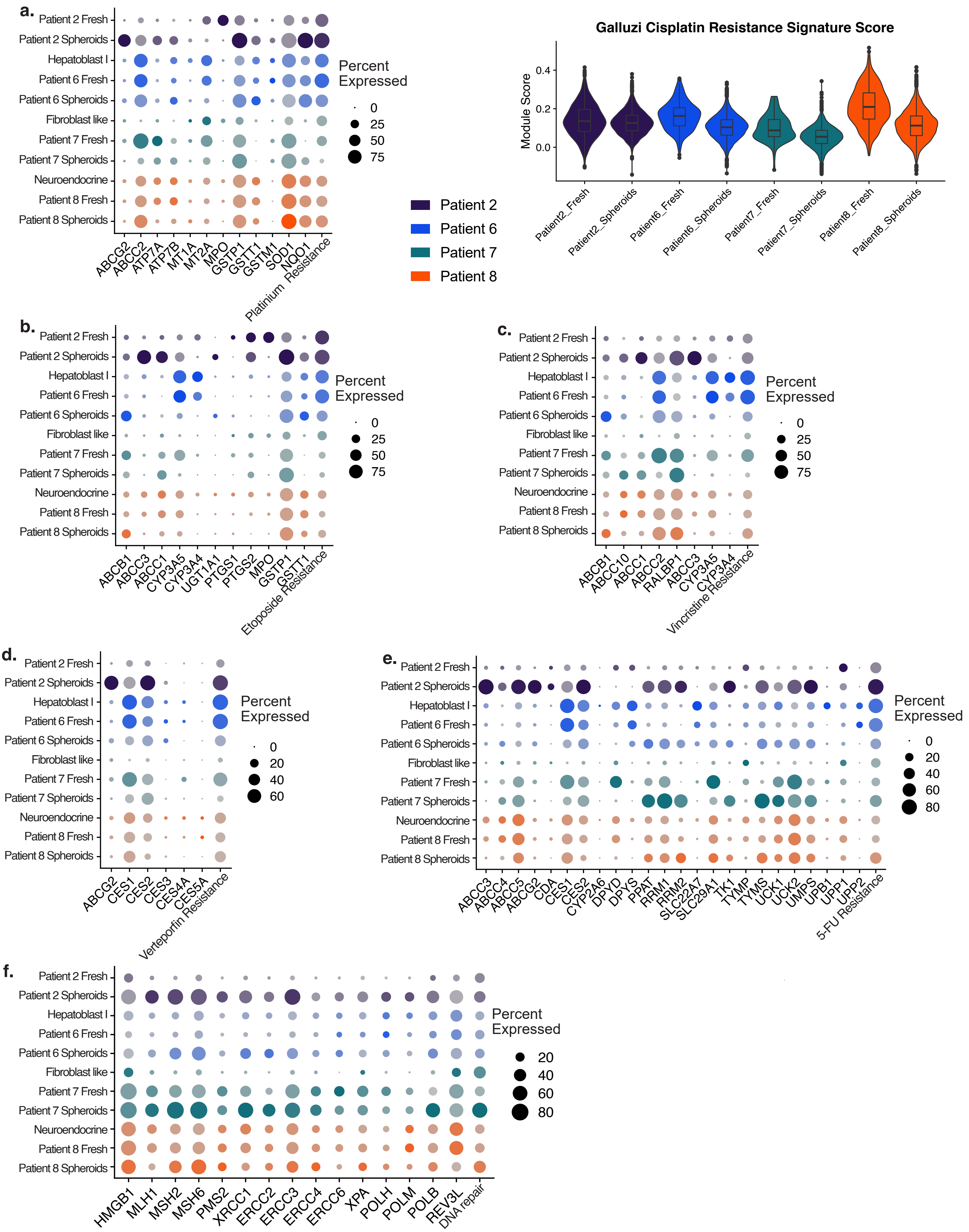

Supplementary Figure 22. Expression of genes involved in drug metabolism and efflux in PDS. Dot plots of expression of genes involved in drug metabolism and efflux and corresponding module scores calculated for each PDS, their parent cells and their corresponding tumor signature, where applicable. a. Platinum-based compounds resistance, and module score for cisplatin resistance signature (Galluzzi et al. 2012) for PDS and corresponding freshly isolated cells, b. Etoposide resistance, c. Vincristine resistance, d. Verteporfin resistance, e. 5-Fluorouracil (5-FU) resistance, and f. DNA-repair. Source data for signature scores are provided as a Source Data file. The box plots present the 25th percentile, the median and the 75th percentile, and outlying or extreme values. The whiskers of the box plots extend to a maximum of 1.5 times the size of the interquartile range. N = 18,859 cell for all plots.
